# Reverse engineering the Gut-Brain Axis and microbiome-metabolomics for symbiotic/pathogenic balance in neurodegenerative diseases

**DOI:** 10.1080/19490976.2024.2422468

**Published:** 2024-11-10

**Authors:** Muhammad Usman Munir, Syed Aoun Ali, Ka Hang Karen Chung, Aleksandr Kakinen, Ibrahim Javed, Thomas Paul Davis

**Affiliations:** a Australian Institute for Bioengineering and Nanotechnology, The University of Queensland, Brisbane, Qld, Australia; b Clinical and Health Sciences,University of South Australia, Adelaide, SA, Australia

**Keywords:** Gut-brain axis, microbiome, neurodegeneration, aging, microbial metabolites, Alzheimer’s, Parkinson’s

## Abstract

Deciphering the molecular communications along the gut-brain axis can help in understanding the pathophysiology of neurodegenerative diseases and exploiting the gut microbiome for therapeutics. However, gut microbes and their metabolites have a multifaceted role in mediating both brain physiology and neurodegenerative pathology. There is a lack of understanding of how and when this role is tipped in neurodegenerative diseases and what are those contributing factors, both at local (gut) and distal (neuronal) levels, that drive this imbalance. Here we have reviewed the gut microbiome and its metabolites in the context of the gut-brain axis and summarized how different factors such as gut-microbial diversity, their metabolites, the role of the native immune system and the integrity of gut epithelial and blood-brain barriers are interconnected and collectively define the involvement of gut-microbiome in neurodegenerative pathologies. It also underlines the need for multidisciplinary tools and animal models to simultaneously reflect on many of these factors and to better correlate with clinical observations and data obtained from human biopsies and fecal samples. Harnessing the gut-brain axis will herald a paradigm shift in medicine for neurodegenerative diseases and aging, emphasizing the significance of the microbiome in the broader spectrum of health and disease.

## GRAPHICAL ABSTRACT

**Figure:**
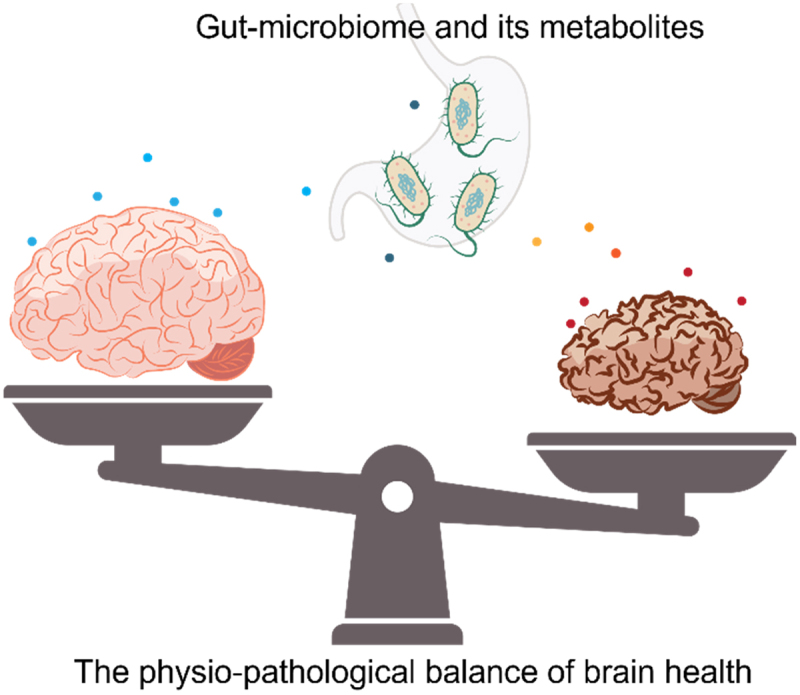

## 1. Introduction

Our understanding of the human-microbiome relationship is evolving with the involvement of multidisciplinary research tools. Spanning numerous disciplines, the gut microbiome’s extensive influence touches upon various aspects of physiology, including the development and functionality of the central nervous system (CNS), specifically the gut-brain axis, an area that is gaining significant traction in the biomedical field. The gut-brain axis is a bidirectional communication pathway that ensures gastrointestinal homeostasis and modulates brain function and behavior.^1,2^ Recent scientific advancements have illuminated the gut’s function in the context of neurodegenerative diseases (NDDs) – disorders characterized by the progressive loss of neuronal function or structure, including conditions such as Parkinson’s disease (PD), Alzheimer’s disease (AD), Huntington’s disease (HD), motor neuron diseases (MND), and various ataxias.^3,4^

The etiology of NDDs is complex, encompassing an interplay of genetics, aging, environmental, and lifestyle elements. Nevertheless, an increasing amount of empirical data indicates that the gut microbiota imbalance, known as dysbiosis, and the resulting disturbance in the composition of microbial metabolites may drive the neuroinflammatory and neurodegenerative mechanisms identified in these disorders.^5,6^ The gut microbiome has been observed to generate a diverse range of bioactive substances that can potentially influence brain physiology. These effects can occur through direct mechanisms, such as permeating across the blood-brain barrier (BBB), impacting the integrity of the gut, i.e., leaky gut, local intestinal inflammation or indirect mechanisms, such as regulating immune responses or neuronal signaling.^7,8^

Gut-microbiome-derived metabolites such as short-chain fatty acids (SCFAs) – including butyrate, acetate, propionate and tryptophan metabolites are of particular interest due to their neuroactive properties and potential to influence the gut-brain axis.^9^ SCFAs, for example, are known to possess anti-inflammatory properties and modulate the BBB integrity.^10^ Similarly, alterations in tryptophan metabolism have been implicated in NDDs processes, highlighting the intricate relationship between metabolic byproducts of the gut microbiota and neurodegeneration.^11,12^ Given the substantial prevalence and the significant burden of NDDs, understanding the potential contribution of gut microbiota to their role in pathophysiology opens a window for novel biomarkers and therapeutic interventions. The modulation of the gut microbiome through diet, probiotics, and prebiotics represents a promising avenue for altering disease progression and possibly developing preventive strategies.^13,14^

**Figure 1.**
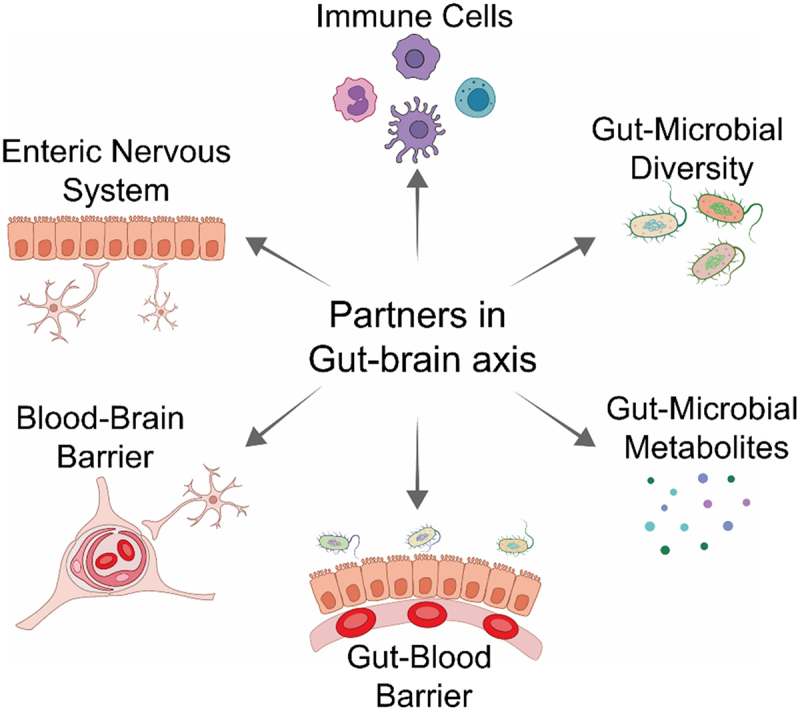
The interacting partners in the gut-brain axis are interconnected and collectively mediate the pathophysiological role of the gut microbiome in the brain health *vs* disease state.

This review aims to explore the role of the gut microbiome in NDDs, with a specific emphasis on the biochemical interactions between microbial metabolites and the pathophysiology of the gut-brain axis. We discussed state-of-the-art gut-microbiome-brain axis research to connect the dots and summarize how the diversity of gut-microbiome, their metabolic flux, pathophysiological integrity of gut-blood and blood-brain barriers and enteric innervations of the GIT (ENS) collectively define the balance of gut-brain axis and influence the development or progression of NDDs (Figure 1). Moreover, this review will highlight the fundamental mechanisms and leverage this understanding to create innovative and impactful treatments to mitigate the effects of these severe conditions.

## 2. Gut-brain axis – the channels of crosstalk between the gut and the brain

**Figure 2.**
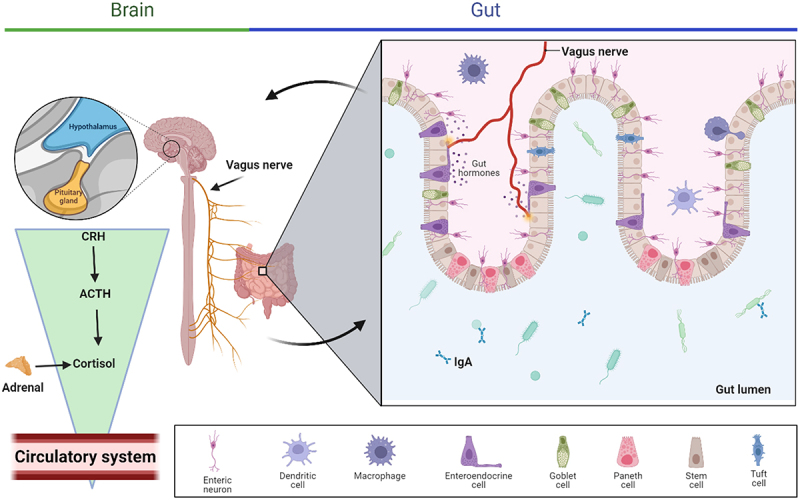
The gut microbiota and the central nervous system (CNS) communicate with each other through various direct and indirect channels of the gut-brain axis. CRH: corticotropin receptor hormone, ACTH: adrenocorticotrophic hormone. Created with BioRender.com

The gut-brain axis is a complex and two-way communication channel that connects the gastrointestinal (GI) system and the CNS. This axis facilitates bidirectional communication between the brain and the gastrointestinal tract, allowing signals to be transmitted from the brain to the gut and vice versa. To fully grasp the impact of the gut on the brain’s health and disease, it is crucial to comprehend the mechanisms by which these conversations take place.^15^ The gut microbiota – brain axis facilitates bidirectional communication between gut bacteria and the brain as represented in Figure 2. This axis plays a vital role in maintaining the homeostasis of GI, CNS and microbial systems.^16^ Most of the understanding of host-microbiota interactions, including the information presented in Figure 2, is derived from animal models that enable control over experimental conditions. The communication pathways encompass the autonomic nervous system, including the ENS, vagus nerve, neuroendocrine system, hypothalamic-pituitary-adrenal (HPA) axis, immunological system, and metabolic ways. The gut microbiota could generate neuroactive substances, including neurotransmitters like GABA, noradrenaline, dopamine, and serotonin (5-hydroxytryptamine (5-HT)), as well as amino acids like tyramine and tryptophan, and microbial metabolites such as short-chain fatty acids (SCFA) and 4-ethylphenylsulfate. These metabolites can go through the portal circulation and interact with the immune system of the host. They can also have an impact on metabolism and affect the local neuronal cells of the ENS and the afferent pathways of the vagus nerve, which directly communicate with the brain.

### 2.1. Vagus nerve is central in gut-brain axis physiology and neuropathology

The enteric nervous system (ENS), commonly known as the “second brain,” is comprised of a complex network of neurons that can operate autonomously from the CNS and vagus nerve is the principal neuronal channel that connects the GI tract with the CNS.^17^ It is essential for gut-brain communications such as influencing the release of neurotransmitters (acetylcholine, gamma-aminobutyric acid (GABA), and serotonin) for mood and anxiety^18^ and distinguishing between symbiotic and pathogenic bacteria through an electrophysiology that needs more investigation. For instance, Tanida et al. demonstrated that injecting *Lactobacillus johnsonii* into the rat duodenum raised the gastric vagal multiunit firing rate by approximately 10% within 15 minutes, eventually reaching a 90% increase an hour after injection.^19^ Certain gut bacteria can produce neurotransmitters and other bioactive molecules that are detectable by vagus nerve and effectively allow the gut microbiota to send signals that can impact mood, anxiety, and overall brain function. For instance, Carabotti et al. studied that the production of serotonin (a neurotransmitter found mainly in the gut and synthesized by gut bacteria) can influence vagus nerve signaling, affecting emotional well-being and gut motility.^20^ From the brain perspective, the vagus nerve is a channel through which it can exert control over gut function. The vagus nerve carries afferent (gut-to-brain) signals crucial for maintaining homeostasis. Sensory neurons of the vagus nerve detect changes in the gut, such as stretching of the stomach walls, and the presence of nutrients and metabolites produced by the microbiota. This information is then relayed to the brain, influencing appetite, satiety, and a host of neuroendocrine responses.^8,21^ Additionally, the vagus nerve is involved in regulating the inflammatory reflexes as reported by Pavlov et al. and upon activation, it could stimulate the spleen’s ability to decrease the secretion of pro-inflammatory cytokines.^22,23^ This pathway known as the “cholinergic anti-inflammatory pathway” illustrates how communication between the gut and the brain through the vagus nerve can regulate immune responses. This mechanism holds potential implications for conditions characterized by inflammation, including specific NDDs.

**Figure 3.**
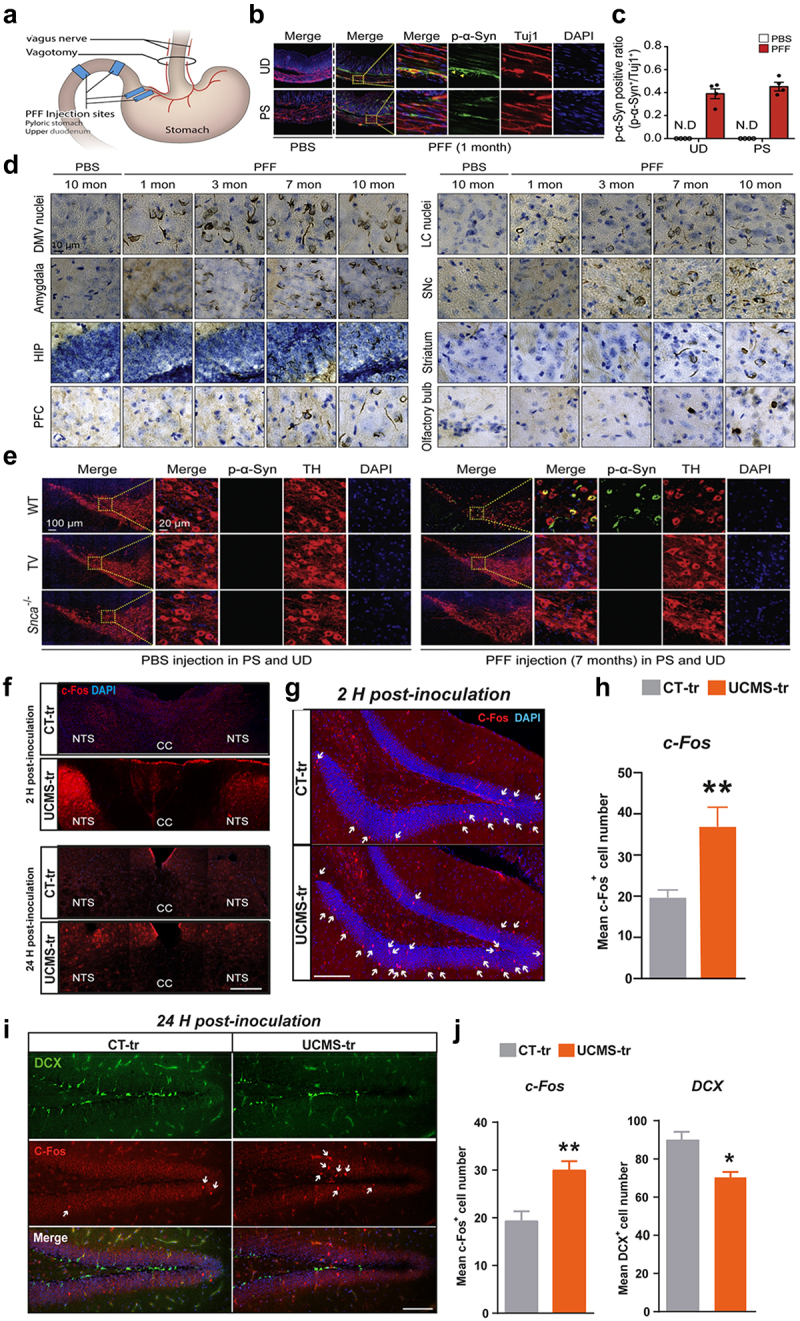
Progression of aSyn pathology in the GI tract and its subsequent spread to the rain. (a) A schematic showing where the aSyn preformed fibrils (PFF) were injected and the site of the vagotomy. (b) Images showing dual staining of pSer129-aSyn (green) and tuj-1 (red) in the upper section of the duodenum and the pyloric part of the stomach, captured one month after the injection. (c) A graphical analysis showing the ratio of pSer129-α-syn-positive neurons to tuj-1-positive neurons in the upper duodenum and pyloric stomach, based on a sample size of four. (d) A depiction of how pSer129-aSyn accumulates in the brains of mice injected with aSyn PFF in the GI tract, showing the spread from the dorsal motor nucleus of the vagus nerve to the olfactory bulb over periods of 1, 3, 7, and 10 months post-injection. (e) The effectiveness of vagotomy and the absence of aSyn in preventing pd-like symptoms following aSyn PFF injection into the GI tract. Illustrated through dual staining for pSer129-aSyn (green) and tyrosine hydroxylase (TH, red) in the substantia nigra pars compacta (SNc). (f) Images showing c-fos immunostaining in the nucleus of the solitary tract (NTS) in mice, following gut microbiota transplantation from either control (ct-tr group) or under chronic unpredictable mild stress (UCMS-tr group) conditions, observed at 2 hours and 24 hours post-transplantation. (g) Comparative images of c-fos immunostaining in the NTS 2 hours after fecal transplantation from control (ct-tr group) and UCMS (UCMS-tr group) microbiota. (h) A graphical representation illustrating the average number of c-fos positive cells in the dentate gyrus at 2 hours after inoculation with microbiota derived from CT and UCMS groups. (i, j) visual representations of C-Fos and DCX immunostaining in the dentate gyrus of mice receiving transplants from ct-tr and UCMS-tr groups. Accompanied by bar charts showing the quantification of positive cells. Panel A-E adapted with permission from cell [25] and panel F-J adapted with permission from nature [26].

In the context of neuropathology, Braak et al. suggested that the pathology of alpha-synuclein (aSyn) might migrate from the GI tract to the brain through the vagus nerve.^24^ This was further confirmed by Kim et al. using an innovative mouse model designed to mimic the transmission of αSyn from the gut to the brain as shown in Figure 3a-e.^25^ In this model, mice received injections of pathogenic aSyn-preformed fibrils into their duodenal and pyloric muscular layers. The progression of aSyn pathology in the brain was tracked, beginning with the phosphorylation of serine 129 at aSyn. Initial signs were detected in the dorsal motor nucleus, followed by the lower regions of the hindbrain, including the locus coeruleus, and eventually reaching areas like the basolateral amygdala, dorsal raphe nucleus, and substantia nigra pars compacta. Correspondingly, the loss of dopaminergic neurons and both motor and non-motor symptoms developed over time. Performing a truncal vagotomy or inducing aSyn deficiency in mice prevented the spread of aSynucleinopathy from the gut to the brain, along with the subsequent neurodegeneration and behavioral issues. This study lends support to the Braak hypothesis regarding the origins of idiopathic PD.

Siopi et al. proposed that the vagus nerve might be a conduit for transmitting the impact of stress-induced alterations in the gut microbiome to the plasticity and behavior associated with the adult hippocampus (HPC) as presented in Figure 3F-J.^26^ Their methodology involved using fecal matter from mice exposed to unpredictable chronic mild stress (UCMS) to inoculate healthy mice. This process was followed by evaluating anxiety and depression-like behaviors, conducting histological and molecular assessments of adult HPC neurogenesis, and examining neurotransmission pathways and neuroinflammation. Specifically, they investigated the vagus nerve’s role in the influence of microbiome alterations on brain function and behavior, employing mice that underwent subdiaphragmatic vagotomy before gut microbiota transfer. The study found that introducing gut microbiome from UCMS mice into healthy mice triggered activation of the vagus nerve, leading to early and persistent alterations in serotonin and dopamine neurotransmission in the brainstem and HPC. These alterations were linked with immediate and lasting impairments in adult HPC neurogenesis and the onset of neuroinflammatory responses in the HPC. Notably, performing a vagotomy negated the deficits in adult HPC neurogenesis and neuroinflammation, as well as depressive-like behavior, indicating that the vagal afferent pathways are essential for the gut’s influence on brain function.

### 2.2. Other enteric nervous systems

The two major components of ENS are the primary plexuses, i.e., the myenteric plexus predominantly governs gastrointestinal motility while the submucosal plexus regulates the gastrointestinal tract’s enzyme secretion and blood flow. These mechanisms collectively empower the ENS to independently manage the intricate processes of digestion, absorption of nutrients and waste disposal. Physiologically, a notable convergence exists in the neurotransmitter systems employed by both the ENS and the CNS, including serotonin, dopamine, and acetylcholine. The existence of a common chemical language enables efficient and uninterrupted communication between the two systems. Notably, approximately 95% of the serotonin in the human body is located in the GI tract. This neurotransmitter is generated by enterochromaffin cells, and its activity is influenced by microbiota. Yano et al. discovered that these interactions have significant implications for the regulation of mood and the potential development of diseases such as irritable bowel syndrome (IBS).^27,28^ In this study, they also demonstrated that the microbiota enhances the production of 5-HT from colonic enterochromaffin cells in a way that can be initiated after birth and reversed. Microbes that form spores, specifically from the microbiota of healthy humans and mice, are effective in modulating 5-HT levels in the serum, colon, and feces. The researchers discovered that certain metabolites, increased by these spore-forming microbes, likely interact directly with colonic enterochromaffin cells to stimulate the production of 5-HT. Crucially, alterations in colonic 5-HT levels due to the microbiota have an impact on GI motility and blood clotting in the host. This suggests that targeting the microbiota could be a viable method for adjusting the availability of peripheral 5-HT and addressing symptoms of diseases related to 5-HT.

The literature indicates that dysfunction within the ENS might contribute to a variety of gut-brain axis disorders, including functional and inflammatory bowel diseases, obesity, and even CNS disorders such as PD. The discovery of aSyn and its intracellular aggregates (Lewy bodies), within the ENS provides compelling evidence for the interconnectedness of these two neural entities. It suggests that neurodegenerative processes might begin in the gut. Braak et al. provide findings showing the presence of aSyn immune-reactive clumps in the gastric myenteric and submucosal nerve networks in samples categorized based on brain pathology associated with sporadic Parkinson’s Disease (sPD).^29^ The observation of these protein accumulations just below the stomach’s epithelial layer suggests that the changes in Meissner’s submucosal plexus might mark the start of a continuous chain of projecting neurons connecting the ENS to the brain’s cerebral cortex.

### 2.3. Non-innervated pathways: systemic circulation and immune system

Communication between the gut and the brain through the circulatory system, independent of direct neural innervation, involves a complex interplay of hormones, immune signals and gut-derived metabolites. This complex signaling network shows the importance of peripheral systems in modulating brain function and behavior, highlighting potential new avenues for therapeutic intervention in neurological and psychiatric disorders. Latorre et al. discussed that the gut endocrine system consists of a wide range of enteroendocrine cells that release over 20 distinct hormones into the bloodstream upon dietary intake. Hormones such as peptide YY (PYY), glucagon-like peptide-1 (GLP-1), and cholecystokinin (CCK) exert significant influence on the regulation of hunger, maintenance of glucose balance and transmission of signals related to satiety to the brain. The impact of these hormones on the hypothalamus and brainstem nuclei, which play a vital role in regulating energy balance, demonstrates a non-neural communication pathway between the gastrointestinal tract and the CNS.^30^

The gut-associated lymphoid tissue (GALT) is the largest collection of immune cells associated with the gut and it serves as a crucial venue for the reciprocal communication between the host immune system and the microbiota residing in the GI tract. Cytokines and chemokines that are generated inside the GALT can access the systemic circulation. Once in circulation, they can communicate with the CNS through two primary pathways: activation of the vagus nerve or passage across the BBB. This passage across the BBB can occur through active transport mechanisms or by utilizing circumventricular organs devoid of a BBB. This immune-to-brain communication can influence neuroinflammatory pathways and has been implicated in the pathophysiology of several neuropsychiatric disorders.^4^

## 3. Gut microbiome: diversity and dynamics

**Figure 4.**
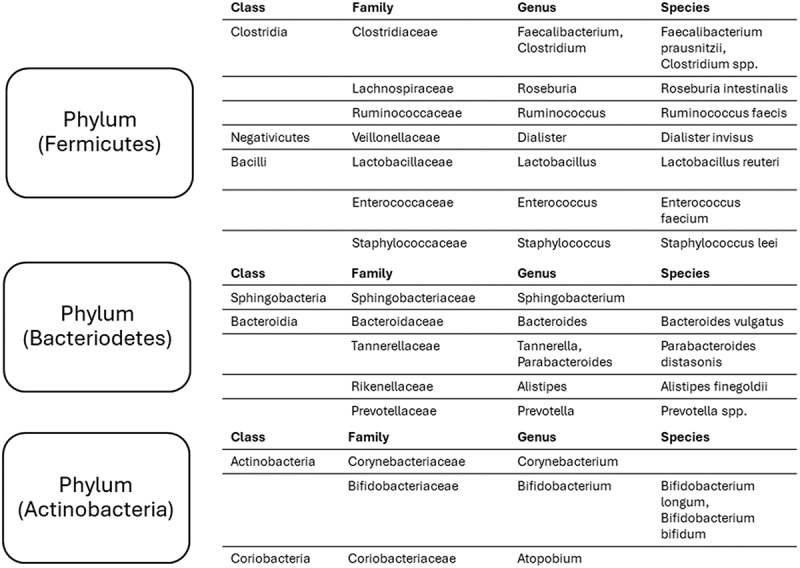
The composition of human gut microbiome. These bacteria represent around 90% of the gut microbiome.

The gut microbiome has a high degree of diversity, encompassing a multitude of bacterial species, among which *Firmicutes* and *Bacteroidetes* constitute the predominant populations. Additional phyla, namely *Actinobacteria*, *Proteobacteria*, and *Verrucomicrobia*, are also observed in the microbial community (Figure 4), though in lower proportions.^31^ The presence of diverse microbial communities in the gut is of utmost importance for preserving gut health. These microorganisms play a crucial role in various physiological processes such as digestion, vitamin synthesis, and maintaining the protective mucosal barrier. Qin et al. conducted an Illumina-based metagenomic sequencing study, assembling and analyzing 3.3 million unique microbial genes from 576.7 gigabases of sequence data obtained from fecal samples of 124 European subjects.^32^ This gene collection, about 150 times larger than the human genome, predominantly includes the more common microbial genes found in the study group and likely represents a significant portion of the typical human intestinal microbial genes. Notably, over 99% of these genes are of bacterial origin, suggesting that the entire group carries between 1,000 and 1,150 common bacterial species, with each hosting at least 160 of these species, which are also largely shared.

Dominguez-Bello et al. studied that the initiation of the gut microbiome commences throughout the process of birth.^33^ They found that babies born through vaginal delivery tend to have a collection of bacteria similar to those found in their mother’s vagina, primarily including types like *Lactobacillus*, *Prevotella*, and *Sneathia*. This implies that the unique mix of vaginal bacteria in each mother is directly passed down to the baby. In most cases of vaginal births (three out of four), the bacteria found in the mother’s vagina closely matched the bacteria in her own baby’s microbiota, more so than the bacteria in other babies born vaginally. On the other hand, babies born *via* Cesarean section (C-section) often have bacteria resembling those typically present on the skin, such as *Staphylococcus*, *Corynebacterium*, and *Propionibacterium*. Unlike vaginal births, the skin bacteria of mothers who had C-sections didn’t show a closer resemblance to their babies than to other babies also born by C-section. This indicates that the mode of birth plays a significant role in determining the initial types of bacteria that colonize a newborn’s body. The diversity of bacterial communities in different body habitats of mothers. It shows the distinct bacterial assemblages in various body parts of mothers, highlighting how each habitat – the oral cavity, skin, and vagina – harbors unique bacterial groups. Furthermore, it details the predominant bacteria in each area, such as *Streptococcus* in the oral cavity, a mix of *Staphylococcus*, *Corynebacterium*, and *Propionibacterium* on the skin, and *Lactobacillus* or *Prevotella* in the vaginal area, demonstrating the specialized nature of bacterial communities in different body regions.

Factors that influence the diversity of gut-microbiome composition predominantly include diet, medications and aging. Chitosan in the diet increases the presence of *Bacteroides*, a group of bacteria known for their ability to break down complex carbohydrates into SCFAs.^34,35^ Chitosan also appears to support the growth of beneficial probiotics while inhibiting harmful bacteria like *Enterobacteria* and *Enterococcus*.^36^ High-sugar diets have been linked to increased *Proteobacteria*, which are often associated with inflammation, and decreased *Bacteroidetes*, which are known for their beneficial roles in gut health.^37^ Anticancer drugs are known to affect the composition of gut bacteria. Li et al. reported that 5-FU treatment led to a reduction in the richness and diversity of the gut bacterial community, causing a decrease in the abundance of *Firmicutes* and a lower *Firmicutes*/*Bacteroidetes* ratio in the feces and cecum.^38^ Additionally, 5-FU decreased the proportion of *Proteobacteria*, *Tenericutes*, *Cyanobacteria*, and TM7 bacteria, while increasing the presence of *Verrucomicrobia* and *Actinobacteria* in these areas. The diversity of the microbiome has been observed to diminish with aging, potentially playing a role in age-related health losses. Bosco et al. studied that age-associated dysbiosis in human beings, termed here as microbiome-aging, is marked by a decrease in *Clostridiales* and *Bifidobacterium* populations, coupled with an increase in *Proteobacteria* and a higher presence of potentially harmful bacteria like *Enterobacteriaceae*.

## 4. Microbial metabolites and their pathophysiological significance

### 4.1. Short-chain fatty acids

Short-chain fatty acids are important microbial metabolites that significantly impact the physiology of the host organism. In the gut, over 95% of Short-Chain Fatty Acids (SCFA) are made up of acetate, propionate, and butyrate, as stated by Van *et al*.^39^ Other SCFAs, such as valerate, iso-valerate, valproate, caproate, isocaproate, succinate, iso-butyrate, and hexanoate, are present but in smaller amounts.^40^

**Figure 5.**
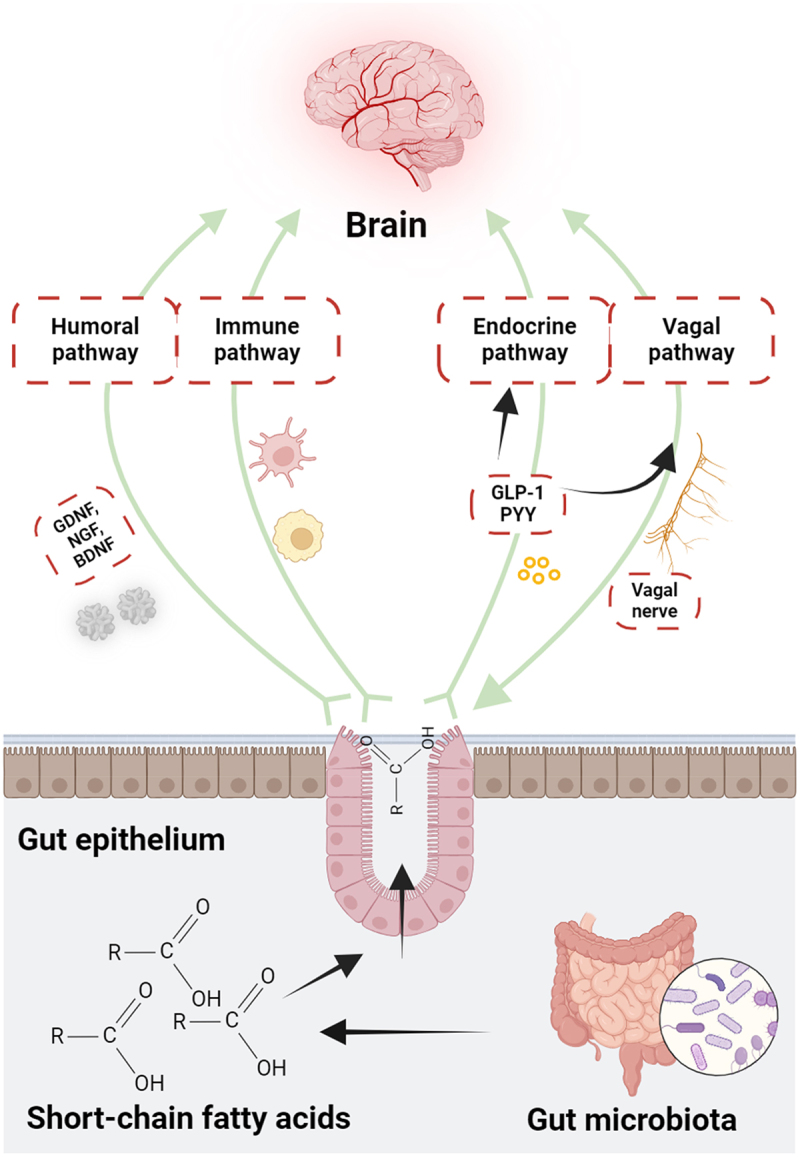
Illustration of the possible routes *via* which short-chain fatty acids (SCFAs) can influence brain function. These acids are taken up by colonocytes and other cells using either H±dependent or sodium-dependent monocarboxylate transporters, or by binding to G protein-coupled receptors. Once inside cells, SCFAs can either inhibit histone deacetylases, leading to more active chromatin and gene expression, or boost histone acetyltransferase activity, resulting in histone acetylation and gene expression. SCFAs affect the gut-brain axis and brain function through diverse pathways including humoral, immune, endocrine, and vagal routes. Through the humoral path, SCFAs can traverse the BBB using monocarboxylate transporters on endothelial cells, influencing the barrier’s integrity by increasing tight junction protein expression. Immunologically, SCFAs impact gut mucosal immunity by activating FFARs or hindering histone deacetylation. They strengthen intestinal barrier integrity by upregulating tight junction proteins and increasing transepithelial electrical resistance. SCFAs also regulate immune cells such as neutrophils, dendritic cells, macrophages, monocytes, and T cells, maintaining bodily balance. Endocrinologically, SCFAs interact with receptors on enteroendocrine cells, triggering the release of hormones like GLP1 and PYY, which signal the brain indirectly via systemic circulation or vagal pathways. They also directly signal the brain through the vagal route. Created with BioRender.com

According to the current literature, SCFAs can influence the brain by direct impacts on body fluids, and indirect effects on hormones, immunological pathways, and neuronal pathways.^41^ They can also impact psychological function by interacting with G-protein-coupled receptors or histone deacetylases as shown in Figure 5. SCFAs, which are produced by gut microbiota, are absorbed by colonocytes and other available cells through H±dependent monocarboxylate transporters (MCTs) or sodium-dependent monocarboxylate transporters (SMCTs). Alternatively, they can bind to G protein-coupled receptors (GPCRs) such as free fatty acid receptors 2 and 3 (FFAR2 and FFAR3), as well as GPCR109A and GPCR164. Intracellular SCFAs could hinder the function of histone deacetylases, inhibiting the removal of acetyl groups from histones. This leads to the formation of chromatin which is more active in transcription. Alternatively, SCFAs can enhance the activity of histone acetyltransferases, causing the addition of acetyl groups to histones and promoting gene expression.

### 4.2. Tryptophan metabolites

Tryptophan (Trp), a vital amino acid, can undergo metabolism by gut bacteria, resulting in the formation of several chemicals that have substantial consequences for human health. A particular mechanism involves the enzymatic conversion of tryptophan into indole and its derivatives. These compounds have been demonstrated to enhance the integrity of the epithelial barrier, regulate the immunological response, and exert effects on the CNS. Zelante et al. have outlined a metabolic process where these Trp metabolites, derived from gut microbiota, regulate mucosal reactivity in mice. In scenarios where Trp becomes the primary energy source instead of sugar (such as when Trp is abundantly available), there’s an expansion of highly adaptive lactobacilli that produces a compound called indole-3-aldehyde. This compound acts as a ligand for the aryl hydrocarbon receptor (AhR) and aids in the AhR-dependent transcription of IL-22 that helps maintain a balanced mucosal response. This balance is crucial for the survival of diverse microbial populations while simultaneously offering resistance against *Candida albicans* colonization and protecting the mucosa from inflammation.

There is substantial evidence suggesting a correlation between the modulation of immunological tolerance and the pathogenesis of various disorders, including depression and neurodegenerative conditions, through the involvement of kynurenine (Kyn) and other metabolites of Trp.^11^ Elevated levels of Kyn and increased activity of the enzyme indoleamine 2,3-dioxygenase 1 (IDO1) have been observed not only in inflammatory bowel diseases (IBD) and irritable bowel syndrome (IBS) but also in neurodegenerative diseases such as AD and PD.

In neurodegenerative conditions, the Kyn pathway’s activation leads to the production of several neuroactive metabolites, including quinolinic acid (QA) and kynurenic acid (KYNA), which have been implicated in neuroinflammation and neurotoxicity. QA, for example, is an excitotoxin that can induce oxidative stress and neuronal death, while KYNA has neuroprotective properties and its dysregulation can contribute to cognitive deficits.^42,43^ In AD, increased levels of IDO1 and Kyn have been associated with enhanced amyloid-beta (Aβ) deposition and tau hyperphosphorylation, two hallmark features of AD pathology. The pro-inflammatory environment in the brain, partly driven by peripheral and central IDO1 activity, may exacerbate the neurodegenerative process by promoting Aβ aggregation and tau phosphorylation. Similarly, in PD, elevated IDO1 activity and Kyn levels have been linked to dopaminergic neuron degeneration in the substantia nigra. The neuroinflammatory milieu, characterized by increased levels of pro-inflammatory cytokines and microglial activation, can enhance the production of neurotoxic kynurenine metabolites, thereby contributing to neuronal damage and the progression of PD.^44^

Thus, the dysregulation of the kyn pathway in the gut-brain axis underlines the critical role of gut microbiota in modulating neuroinflammatory and neurodegenerative processes. Understanding these mechanisms can open potential therapeutic avenues, such as targeting the kynurenine pathway with specific inhibitors to mitigate neurodegeneration and improve cognitive and motor functions in affected individuals.

### 4.3. Secondary bile acids

Bile acids are derived from cholesterol through hepatic synthesis and subsequently undergo metabolism by the gut microbiota, forming secondary bile acids. These secondary bile acids engage with multiple receptors and signaling pathways, including the farnesoid X receptor (FXR) and the G-protein-coupled bile acid receptor (GPBAR1 or TGR5). These interactions play a crucial role in lipid and glucose metabolism, energy balance, and immune regulation.^45,46^ Moreover, there is a correlation between dysregulation in bile acid metabolism and the occurrence of liver disorders and colorectal cancer.^47^

Recent studies have highlighted the significant impact of bile acid dysregulation on neurodegenerative diseases such as AD and PD. In neurodegenerative conditions, altered bile acid metabolism has been linked to changes in the gut-brain axis that can influence disease progression. For instance, bile acids can cross the BBB and interact with neural cells, thereby modulating neuroinflammatory responses and neuronal survival.^48^

In AD, alterations in bile acid profiles have been associated with Aβ aggregation and tau hyperphosphorylation. Elevated levels of certain bile acids, such as deoxycholic acid (DCA), have been found to correlate with increased neuroinflammation and oxidative stress, which are key factors in AD pathogenesis. Conversely, bile acids like ursodeoxycholic acid (UDCA) have shown neuroprotective effects by reducing Aβ toxicity and improving mitochondrial function.^49^

Similarly, bile acid dysregulation has been linked to the degeneration of dopaminergic neurons in PD. Studies have shown that changes in bile acid metabolism can affect the gut microbiota composition, leading to increased gut permeability and systemic inflammation. This “leaky gut” condition allows pro-inflammatory cytokines and microbial metabolites to enter the bloodstream and reach the brain, where they can exacerbate neuroinflammation and neuronal damage. Bile acids like tauroursodeoxycholic acid (TUDCA) have demonstrated potential in protecting dopaminergic neurons and mitigating PD symptoms by modulating endoplasmic reticulum stress and mitochondrial dysfunction.^50^

Furthermore, bile acids can influence the gut microbiota’s composition and function, creating a feedback loop that impacts both peripheral and central nervous system health. Insights of the precise mechanisms through which bile acids contribute to neurodegeneration could open new beneficial ways for modulating bile acid pathways to treat or prevent NDDs.

### 4.4. Phenolic compounds

These are a varied collection of secondary metabolites becoming more acknowledged for their capacity to nourish the neurological system, specifically concerning AD and PD. The substances mentioned, namely flavonoids, phenolic acids, tannins, and lignans, all play a role in regulating oxidative stress, inflammation, and the functioning of neurons.

Flavonoids, including quercetin, catechins, and anthocyanins, are renowned for their potent antioxidant properties. Their function involves the removal of ROS and the enhancement of the body’s antioxidant defenses. Moreover, flavonoids can regulate signaling pathways that play a vital role in inflammation and cell survival, therefore producing neuroprotective effects. Recent research has shown that these substances have the potential to enhance cognitive performance and decrease neuroinflammation, making them intriguing candidates for treating AD and PD.^51,52^

Phenolic acids, such as caffeic acid, ferulic acid, and chlorogenic acid, also possess strong antioxidant effects. They have the ability to hinder the process of lipid peroxidation and regulate the pathways involved in neuroinflammation by reducing the activity of pro-inflammatory cytokines and enzymes. Phenolic acids have been shown to provide evidence of protection against neuronal damage and enhancement of cognitive deficits in animal models of neurodegeneration.

Tannins, such as ellagitannins and proanthocyanidins, are acknowledged for their antioxidative and anti-inflammatory characteristics. They can form complexes with metal ions, which stops the creation of free radicals and hence safeguards nerve cells from oxidative harm. Empirical research has emphasized the neuroprotective properties of tannins in models of AD and PD, indicating their capacity to potentially decelerate the advancement of both conditions.^53^

Lignans, such as secoisolariciresinol and matairesinol, possess both antioxidant and anti-inflammatory properties. These chemicals can regulate hormonal activity by interacting with estrogen receptors, which can provide defense against neurodegenerative disorders.^54^ The consumption of dietary lignans has been linked to a decreased likelihood of experiencing cognitive decline and neurodegeneration. This is achieved by regulating oxidative stress and inflammation.

### 4.5. Trimethylamine N-oxide

Trimethylamine N-oxide (TMAO) is a compound that is formed in the gut by the oxidation of trimethylamine (TMA). TMA is derived from certain nutrients in the diet, such as choline, carnitine, and betaine. TMAO has attracted considerable interest due to its probable involvement in multiple neurodegenerative disorders, such as AD and PD.^55^

TMAO has the ability to pass through the BBB, and its higher concentrations in the blood have been linked to greater neuroinflammation and oxidative stress, which are important factors in the development of neurodegenerative conditions. Elevated levels of TMAO in the bloodstream have been associated with cognitive decline and the accumulation of Aβ plaques, as well as the excessive phosphorylation of tau proteins, in individuals with AD. These pathogenic characteristics lead to the degeneration of neurons and the advancement of AD.^56^ Increased levels of TMAO have been observed to worsen both neuroinflammation and motor impairment in individuals with Parkinson’s disease. Research conducted on animal models of PD, such as the mouse model caused by MPTP, has shown that TMAO can worsen motor impairments and neuroinflammatory reactions. TMAO specifically enhances the activation of glial cells in the striatum and hippocampus, hence stimulating the release of pro-inflammatory cytokines that cause additional harm to dopaminergic neurons.^57^

Furthermore, TMAO has been linked to the impairment of BBB integrity. This disruption might enhance the penetration of additional detrimental substances into the brain, therefore intensifying neuroinflammatory and neurodegenerative processes. The adverse impact of TMAO on neuronal well-being highlights its potential as a biomarker for early identification and as a focal point for therapeutic intervention in neurodegenerative disorders. By focusing on reducing TMAO levels through changes in diet or the use of drugs, we may have new opportunities to slow down the advancement of diseases such as AD and PD, as TMAO has been found to worsen these ailments.^58^ Additional study is required to completely understand the processes by which TMAO affects neurodegeneration and to create efficient strategies for therapeutic control.

### 4.6. Indoles

Indoles are a group of chemicals produced by gut bacteria through the breakdown of the amino acid tryptophan. They have a notable impact on the connection between the stomach and the brain and have been linked to different neurodegenerative illnesses. Indole and its derivatives, including indole-3-propionic acid (IPA), indole-3-acetate, and indoxyl sulfate, can either have neuroprotective or neurotoxic effects depending on their concentration and the circumstances.^59^

#### 4.6.1. IPA (indole-3-propionic acid)

IPA is a compound that has significant neuroprotective effects. It functions as a powerful antioxidant, eliminating harmful free radicals and decreasing oxidative stress, a significant contributor to the advancement of neurodegenerative disorders. Research has demonstrated that IPA has the ability to hinder the development of Aβ plaques and tau tangles, which are the characteristic features of Alzheimer’s disease. As a result, IPA safeguards neurons against oxidative stress.^60^

#### 4.6.2. Indole-3-acetate and indoxyl sulfate are chemical compounds

These metabolites, albeit originating from tryptophan, can have varying impacts. Indole-3-acetate has been demonstrated to affect the integrity of the intestinal barrier and regulate immunological responses. At elevated levels, Indoxyl sulfate can have a neurotoxic effect, leading to inflammation and oxidative stress in the brain. Increased concentrations of indoxyl sulfate have been associated with cognitive decline and worsening of neurodegenerative disorders including Parkinson’s disease.

Indoles influence the communication between the gut and the brain through many methods. These substances could pass through the blood-brain barrier and have a direct impact on brain cells. They can also influence the creation of neurotransmitters like serotonin and regulate the immune system by interacting with AhR in both the stomach and brain.^61^ These interactions have the potential to induce alterations in neuroinflammation, neuronal well-being, and general cognitive performance.

Recent investigations have provided additional clarity on the involvement of indoles in the process of neurodegeneration.^62^ Research has demonstrated that indoles can reduce neuroinflammation by decreasing the production of pro-inflammatory cytokines and increasing the activation of anti-inflammatory pathways. Furthermore, maintaining a delicate equilibrium between helpful and detrimental indole metabolites is essential for preserving the health of neurons. This implies that adjusting the composition of gut microbiota to promote the production of protective indoles could be a viable therapeutic approach.

### 4.7. Microbial amyloids

Microbial amyloids are self-assembled nano-fibers of proteins made by bacteria and they are analog in structure to the pathogenic amyloids of Aβ and aSyn.^63^ As the gut is the largest reservoir of microbes, it is hypothesized that microbial amyloids from the gut can access extra-intestinal tissues such as the brain and have a role in AD pathology or overall aging.^64^ We have demonstrated for the first time on this hypothesis that FapC amyloids made by *P. aeruginosa* can accelerate the fibrilization and exacerbate the toxicity of Aβ triggering the early onset of AD.^65^ Similarly, Matthew Chapman’s team has demonstrated CsgA amyloids by *E. coli* can exacerbate aSyn fibrilization.^66^ Given the biophysical aspects of feasible interaction between microbial amyloids and pathogenic amyloids, the next question is whether there are amyloids in the gut and how they can make their way to the brain. It has been recently demonstrated that the human gut microbiome has at least 30 genes (*in silico* analysis) that can produce proteins with a sequence similar to the amyloidogenic sequence of *Staphylococcus aureus* only.^67^ Computational analysis showed these sequences can self-assemble into amyloids and further analysis of human fecal samples showed the presence of amyloid structures. These amyloid structures isolated from human fecal samples triggered aSyn aggregation and Parkinson’s symptoms in PD mice models, upon cerebral injection. Considering the shared cross-β sheet architecture of microbial amyloids and prion amyloids, it is reasonable to hypothesize that microbial amyloids can propagate from the gut to the brain in a prion-like mechanism through the enteric nervous system. This has also been shown in the context of aSyn where transgastric propagation of aSyn amyloids was observed in mice and this propagation was inhibited upon vagotomy, indicating a role of the enteric nervous system in facilitating gut-to-brain propagation of amyloid-like structures to provide a potential window for them to participate in NDDs. Microbial amyloids are majorly produced by microbes to support their biofilm structure. We have recently discovered an anti-biofilm and anti-microbial amyloid role of Aβ peptide where stoichiometric low concentrations of Aβ peptide were able to remodel microbial amyloids and disintegrate the biofilms made by microbial culture.^68^ This resulted in sensitizing the underlying microbial cells to lower concentrations of antibiotics. Considering this anti-biofilm physiology of Aβ, repeated or sustained exposure to microbial biofilms either through gut dysbiosis or through direct infection in the central nervous system can also lead to Aβ production beyond a level that can be managed by proteostasis and thus leading to AD pathogenesis.

## 5. Gut microbiome in neurodegenerative diseases

### 5.1. Alzheimer’s disease

Alzheimer’s disease (AD) is a most common neurodegenerative condition predominantly characterized by the progressive loss of cognitive functions, primarily affecting memory, spatial navigation, and executive functions. The accumulation of Aβ plaques and neurofibrillary tangles (Tau) within the brain histologically mark AD pathogenesis.^69^ Dodiya et al. suggested shifting the focus toward understanding the gut microbiome’s role in AD pathophysiology and postulated a critical connection between microbial dysbiosis and neuroinflammatory pathways involved in the disease. This is based on the observations that in individuals with AD, the gut microbiome often exhibits a distinctive compositional shift. There tends to be an increase in the abundance of pro-inflammatory bacterial genera such as *Bacteroides*. These microbes are known to influence the gut’s permeability and may contribute to the systemic and central inflammation observed in AD by facilitating the translocation of lipopolysaccharides (LPS) and other inflammatory mediators into the bloodstream.^70^

Conversely, there is a notable reduction in anti-inflammatory and neuroprotective species, like those belonging to the genus *Faecalibacterium*. These bacteria are prolific producers of butyrate, a short-chain fatty acid with potent anti-inflammatory properties, essential for maintaining gut barrier integrity and modulating immune responses. Lower levels of butyrate-producing bacteria have been correlated with increased gut permeability, a phenomenon commonly referred to as “leaky gut,” and a higher systemic inflammatory state, both of which have been associated with the cognitive decline seen in AD.^71^

This shift toward a pro-inflammatory microbial milieu may exacerbate the neuroinflammation inherent in Alzheimer’s pathogenesis. Pro-inflammatory cytokines, potentially stemming from microbial imbalance, can cross the compromised BBB and activate microglia, the brain’s resident immune cells. Once activated, these cells can perpetuate the cycle of neuroinflammation and neurodegeneration by producing inflammatory mediators and reactive oxygen species, further contributing to the pathology of AD.^72^ Jiang et al. have reviewed studies indicating that germ-free animals, as well as those exposed to pathogenic microbes, antibiotics, probiotics, or fecal microbiota transplants, show evidence of the gut microbiota’s influence on host cognitive functions and the development of AD. They suggest that the disruption in the microbiota can lead to increased permeability of both the gut-blood and the blood-brain barrier, which might play a role in the onset or progression of AD and other NDDs, particularly those linked to aging. Moreover, the gut microbiota’s bacterial population can produce significant amounts of amyloids and lipopolysaccharides. These substances are thought to affect signaling pathways and stimulate the release of pro-inflammatory cytokines, which are involved in the AD pathogenesis.^73^

Additionally, the gut microbiome’s altered state in AD could influence the disease course through its impact on the metabolism of amyloid precursors and tau phosphorylation, thereby affecting the aggregation of amyloid plaques and the formation of neurofibrillary tangles, hallmark features of Alzheimer’s pathology.^74^ Under this hypothesis, the microbial dysbiosis can induce an increase in generalized reactive oxygen species generation, that activate NF-kB and proinflammatory mRNA-34a, eventually downregulating TREM-2 receptors on microglial cells. TREM-2 receptors are involved in microglial/myeloid cells recognition of misfolded/aggregated proteins and therefore an impairment in its function leads to the accumulation of Tau and Aβ plaques. Given the associations between microbial dysbiosis and AD, there is growing interest in modulating the gut microbiome as a potential therapeutic strategy.^75^ The administration of specific probiotics, prebiotics, or a combination known as synbiotics could help restore microbial balance, reduce inflammation, and ultimately impact disease progression. The efficacy of such interventions in altering the course of AD remains an active and promising area of research.^76^ In brief, the alterations in microbial composition found in individuals with AD, which are marked by an augmentation in inflammatory species and a reduction in protective ones, provide valuable insights into possible treatment targets and biomarkers for AD pathology.^77,78^

#### 5.1.1. Gut microbiome-derived metabolites and AD

Owing to the complexity of gut-microbial ecosystem, an imbalance in microbial metabolites has been implicated in the neuropathological manifestations that are commonly observed in AD. These manifestations include the deposition of Aβ plaques and phosphorylation of Tau, which play crucial roles in the onset and progression of AD. The accumulation of Aβ peptide in the brain, which leads to the formation of plaques, is a leading hypothesis for AD therapeutics, i.e., monoclonal antibodies such as Donanemab that have shown cognitive improvement in early AD.^79^ Microbial metabolites, specifically SCFA, have been observed to exert an influence on immunological responses and possess the potential to regulate inflammation inside the CNS, hence altering the pathophysiology of Aβ. Research findings have indicated that specific gut microbiota can modify the composition of bile acids, thereby impacting the solubility and clearance of Aβ. This, in turn, can influence the formation of plaques.

**Figure 6.**
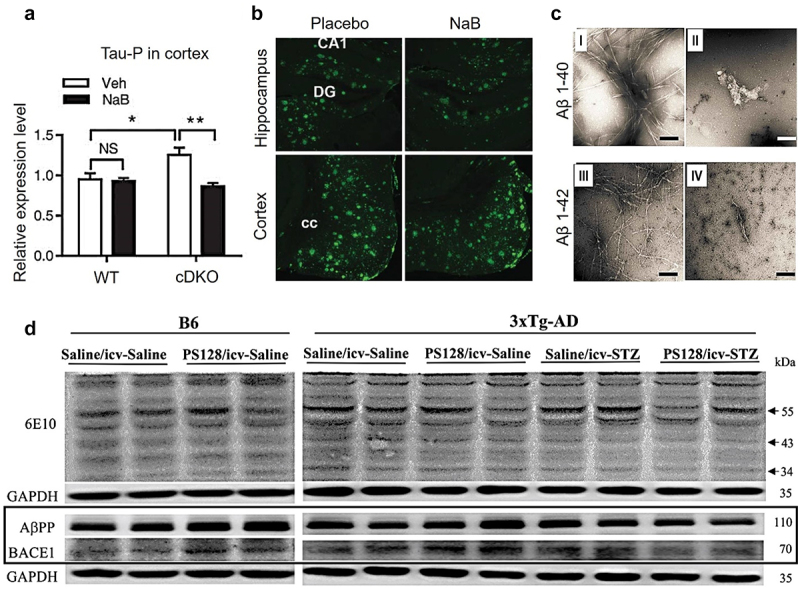
Neuroprotective effect of SFCA in AD: sodium butyrate (NaB) reduced the levels of phosphorylated tau in the cortex of forebrain presenilin-1 and presenilin-2 conditional double knockout (cDKO) mice, in contrast to the vehicle (Veh) control (a). In 15 months, old APP/PS1 mice with an advanced stage of AD disease and Aβ burden, no reduction in Aβ burden was observed with NaB treatment (b). As compared to the vehicle control (panel I, III), valeric acid (panel II, IV) inhibited fibrillar aggregation of Aβ by direction interactions. Scale bars indicate 100 nm (c). In 3xTg-ad mice, intracerebroventricular (icv) injection of streptozotocin (STZ) induced AD histopathology, specifically higher expression of AβPP and BACE1 that was mitigated with oral gavage of lactobacillus plantarum PS128 (d). Panel A, B, C, and D were adapted with permissions from frontiers [59], IOS press [60], Taylor & Francis [62], and BMC [63], respectively.

In AD, the Tau, which plays a role in stabilizing microtubules in neurons, undergoes hyperphosphorylation. This post-translational modification of Tau contributes to the development of neurofibrillary tangles. Empirical evidence indicates that metabolites produced by gut microbes can traverse the BBB and engage with CNS pathways responsible for regulating Tau phosphorylation. Indoles that are produced through the metabolic breakdown of Tryptophan by gut bacteria have demonstrated neuroprotective characteristics and the potential to influence the dynamics of tau phosphorylation.^75^ In presenilin-1 and presenilin-2 conditional double knockout (cDKO) mice models, treatment with sodium butyrate (intraperitoneally, 1.2 g/kg body weight dose once a day for 21 days) improved contextual memory and reduced Tau hyperphosphorylation (Ser-199, Ser-202) and inflammatory glial fibrillary acidic protein (GFAP) (Figure 6a). These neuroprotective effects of sodium butyrate were relatable with the restoration of histone acetylation in cDKO mice.^80^ Govindarajan et al., studied the effect of sodium butyrate (1.2 g/kg, once daily for 6 weeks) in double transgenic APP/PS1 mice models (15 months old) where treatment with sodium butyrate didn’t induce any effect in Aβ levels in the cortex and hippocampus of the mice (Figure 6b). However, there was some improvement in the contextual memory of the mice.^81^ No improvement in the histopathology of the mice can be attributed to the advanced stage of the disease at 15 months of age where histopathology was beyond recovery. Fernando et al. provided complementary results with 5XFAD mice of 2 months age, i.e., early stage of the AD.^82^ Sodium butyrate at the dose of 5 and 15 mg/kg/day for 12 weeks resulted in a reduction of Aβ plaque burden from 1619.88 pg/mg of brain tissue (control) to 852.87 and 756 pg/mg of brain tissue, respectively. This was accompanied by an improvement in cognitive function (Cue Fear test) of the mice. These investigations were explained by the hypothesis that the histone acetylation mechanism of SCFA can protect against AD pathology. Ho et al., presented a direct inhibition of Aβ fibrillization, for both Aβ 40 and 42 variants, in the presence of valeric acid,^83^ however, an insignificant effect of aggregation kinetics was observed (Figure 6c). Huang et al., used 6–7 months 3×Tg-AD mice and reversed streptozotocin-induced AD histopathology and cognitive decline through gut-microbiota modulation with SCFA producing *Lactobacillus plantarum* PS128^84^ (Figure 6d). Inoculation of gut-microbiota with PS128 *via* oral gavage for 33 days (100 µL of 10^10^ CFU/mL) reduced AβPP (Aβ precursor protein) and Aβ metabolic enzyme BACE1.

**Figure 7.**
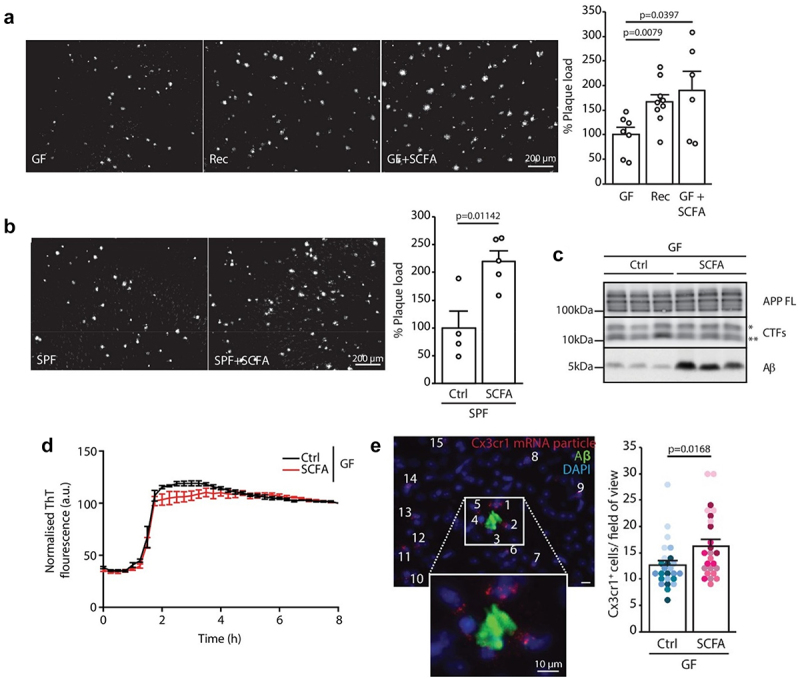
Pathological role of SFCAs in AD: germ-free (GF) APP/PS1 mice presented lower burden of Aβ. However, when GF mice were recolonized (rec) or supplemented with SFCAs (GF + SFCA) they presented a higher Aβ plaque burden (a). Similarly, specific pathogen-free (SPF) mice presented a higher Aβ plaque burden upon SFCA supplementation (b). Western blot analysis (c) presented a high Aβ load with SFCA supplementation. *in vitro* incubation of SFCA with Aβ presented that SFCA do not interfere with Aβ aggregation kinetics (d). Increased microglial recruitment at the site of Aβ plaque in SCFA-supplemented germ-free mice (e). Adapted with permission from eLife science publications [64].

This may indirectly represent a reduced Aβ burden in the brain in relation to SCFA, however, results from Colombo et al., indicate otherwise.^85^ Based on the discussion above, indicating a neuroprotective effect of gut microbial-derived SCFA, Colombo et al., included the role of microglial cells and observed an increased Aβ cerebral burden when germ-free APP/PS1 transgenic mice were supplemented with SCFA (through drinking water). Germ-free and specific pathogen-free mice presented a lower Aβ burden and plasma SCFA levels, which upon supplementation with SCFA or recolonization of germ-free mice led to higher Aβ plaques (Figure 7a, b). Western blots showed a higher Aβ burden in SFCA-supplemented mice (Figure 7c) while ThT assay didn’t present any difference in fibrilization kinetics of Aβ in SCFA presence (Figure 7d). The role of SCFA in increasing Aβ plaques burden was explained based on higher microglial activation and recruitment at the site of Aβ plaques in SCFA-supplemented germ-free mice (Figure 7e). In APP/PS1 transgenic mice, both butyric and isobutyric acid levels were reduced in feces and brain, with a positive correlation between brain and fecal butyric acid levels as studied by Zhang *et al*.^86^ Another study using stable isotope labeling and liquid chromatography-tandem mass spectrometry found lower propionic acid and higher lactic acid levels in APP/PS1 mice compared to wild-type mice.^87^ Syeda et al. observed that the discrepancies in SCFA concentrations in AD and wild-type mice were present at various ages and life stages. For instance, 11-month-old 3×Tg-AD mice showed a more significant decrease in SCFA than younger 3×Tg-AD mice, a pattern emerging later in wild-type mice.^88^ Additionally, AD-model Drosophila exhibited a notable reduction in acetate concentrations, alongside diminished *Acetobacter* and *Lactobacillus* populations.^89^ This evidence for the role of gut-microbial derived SCFA can be summarized into an understanding that SCFA can have a role in protecting AD-related histopathology, specifically phosphorylated Tau, in the early stages of AD mice models. However, they didn’t show any promise in mitigating the histopathology in late-stage AD or by directly inhibiting Aβ fibrilization. Also, when considering the role of microglial cells, SCFA increased AD pathology and Aβ plaque burden in mice models through microglial activation and recruitment at the site of Aβ plaque. This indicates a multifaceted paradigm of AD pathology in relation to gut-microbiome-derived SCFA.

In terms of human data, differences are observed in the diversity of gut microbiota in both AD patients and animal models, affecting the levels of SCFA.^90^ In a study by Yilmaz et al., saliva samples from AD patients, analyzed using^1^H-NMR metabolomics, showed higher propionate levels compared to healthy individuals.^91^ Another study by Figueira and coworkers found increased levels of propionate and acetic acid in saliva from AD patients, being 1.35 and 1.25 times higher, respectively, than in controls.^92^ Conversely, Cui et al. noted lower acetate levels in AD patient serum, correlating with an intensified AD risk.^93^ In fecal samples, decreases in seven SCFA, including formic, acetic, propionic, butyric, 2-methylbutyric, isovaleric, and valeric acids, were observed across healthy controls, those with amnestic mild cognitive impairment, and AD patients.^94^

Other microbial metabolites that are emerging to have a strong correlation with Aβ aggregation and AD pathology are microbial amyloids and LPS. Similar to Aβ, certain opportunistic gut pathogens secret specific proteins that self-assemble into similar amyloid-like structures and provide a scaffold for microbial biofilm formation. Examples include FapC from *Pseudomonas* and CsgA from *E. coli*.^95^ The literature indicates the role of LPS and microbial amyloids in Aβ induced AD pathology through a combination of different mechanisms such as reactive oxygen species generation, innate immune activation, neuroinflammation and feasibility of direct molecular mimicry that can facilitate cross-seeding.^96^ Our team presented evidence of direct interaction between LPS and microbial amyloids of FapC from *Pseudomonas aeruginosa* that cross-seed the aggregation of Aβ into neurotoxic species and participated in the pathogenesis of AD.^65,97^ In zebrafish models of Aβ induced AD-like pathology, the introduction of seeds from FapC amyloids, triggered an early onset and accelerated the cognitive decline in zebrafish larvae and led to higher histopathological deterioration.

The investigation of microbial metabolites and their correlation with AD presents unique opportunities for comprehending the pathology of the disease. It emphasizes the prospects for innovative therapeutic interventions that focus on modulating the gut microbiota. Nevertheless, further investigation is required to clarify these intricate associations comprehensively and apply these discoveries effectively in a clinical setting.

### 5.2. Parkinson’s disease (PD)

Parkinson’s Disease (PD) ranks as the second most prevalent neurodegenerative condition that is distinguished by the degeneration of dopaminergic neurons in the substantia nigra region of the brain. This degeneration results in motor control impairments, including tremors, rigidity, and bradykinesia. It mainly impacts older adults, and its occurrence is expected to increase two-fold from 2005 to 2030.^98^ The causes of PD are believed to involve multiple factors, although they remain largely unclear.^99^

A growing amount of research indicates that changes in the gut microbiome may be associated with the development and manifestation of PD. Several studies have identified an over-representation of the *Helicobacter* genus – known for its pathogenic species *Helicobacter pylori*, in individuals with PD. This bacterium has been associated with increased inflammation and has been hypothesized to contribute to the neuroinflammatory processes observed in PD.^100^

Conversely, a reduction in members of the *Prevotellaceae* family, which are typically involved in mucin production and maintaining a healthy gut mucosal barrier, has been reported in PD patients. This leads to enhanced gut permeability, also referred to as “leaky gut,” allowing potentially harmful substances to permeate into the circulatory system and possibly trigger immune responses that could affect the brain.^101^ These observations are consistent with the hypothesis that gut dysbiosis may contribute to the onset and progression of PD *via* several potential mechanisms, such as 1) directly through migrating pathogenic bacterial metabolites from the gut to the brain, 2) indirectly through the modulation of systemic and neuroinflammation which can affect neuronal health, 3) impacting the production and regulation of neurotransmitters, some of which are synthesized in the gut and have been implicated in PD.

Identifying specific microbial patterns in PD has driven the initial interest in the potential for microbiome-targeted therapies, such as probiotics, dietary interventions, and fecal microbiota transplantation as adjuvant treatments for PD.

#### 5.2.1. Gut microbiome-derived metabolites and PD

**Figure 8.**
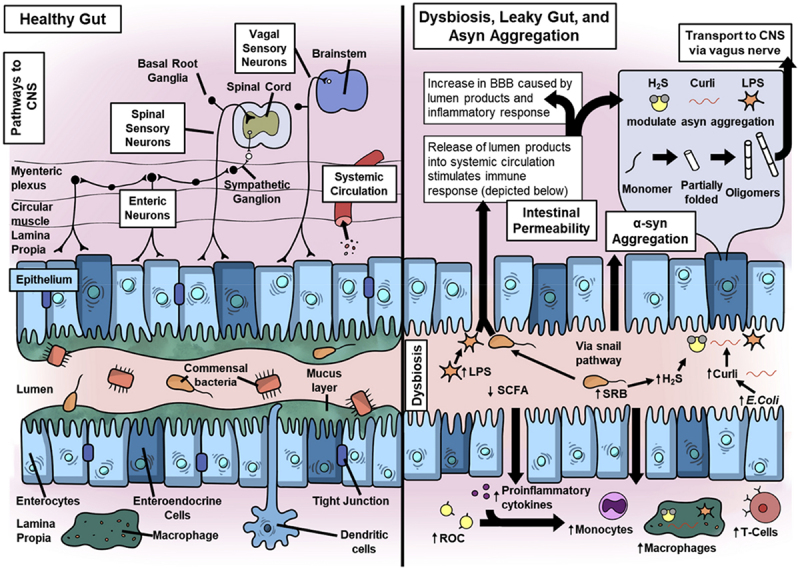
A comparison of healthy and leaky gut in relation to PD pathology triggering from the gut. The healthy equilibrium maintained by commensal microbes and epithelial and immune cells is lost in a dysbiotic/leaky gut. A gut dysbiosis leads to the dominance of LPS, curli and reactive oxygen species (ROC) producing bacteria that (i) increase the permeability of the epithelial barrier and (ii) initiate aggregation of aSyn monomers to oligomers which are proposed to propagate from gut to the brain. These microbial products also activate adaptive and innate inflammatory responses and increase circulatory inflammatory cytokines, ROC, monocytes, macrophages and T cells. Reprinted with permission from Springer [89].

The pathophysiology of PD is closely associated with the misfolding and aggregation of the aSyn protein in the brain, which forms Lewy bodies and leads to neuronal degeneration.^102^ In terms of the involvement of gut bacteria, *Helicobacter pylori (H. pylori)* – a gastric pathogen involved in peptic ulcers, was initially found to correlate with PD where motor symptoms appeared to get worse in association with *H. pylori* infection.^103^ The notion that *H. pylori* infection can lead to PD as a predisposing factor needs investigation. However, there is a strong association between chronic gastric infection of *H. pylori* and exacerbation of motor PD symptoms. The most relevant hypothesis for exacerbation of PD is through *H. pylori* neurotoxins such as vacuolating cytotoxin VacA and cytotoxic associated gene encoding CagA.^104^ Weller et al. predicted a correlation between parkinsonism and CagA immunoblot seropositivity.^105^ Additionally, *H. pylori* infestation of the gut alters the oral pharmacokinetics of levodopa – a medication for PD, through consumption of orally administered levodopa by *H. pylori* in niche competition.^106^ Eradicating *H. pylori* infection improves the patient’s response to levodopa.^107^ Other gut microbiome-derived metabolites, particularly SCFA can also influence PD pathogenesis. Unger et al. found a reduction in SFCA levels, bacterial phylum *Bacteroidetes* and family *Precotellaceae* while abundance of *Enterobacteriaceae* in the fecal samples from PD patients in comparison to age-matched controls.^108^ They conducted a detailed analysis of SCFA concentrations using gas chromatography and assessed the microbiota composition through quantitative PCR in fecal samples from 34 PD patients and 34 age-similar controls. Their findings revealed a significant decrease in fecal SCFA levels, specifically acetate, propionate and butyrate in the fecal samples of PD patients compared to the age-matched healthy control group. Additionally, they noted a reduction in the *Bacteroidetes* phylum and *Prevotellaceae* family, while the *Enterobacteriaceae* family was more prevalent in the fecal samples of PD patients. A significant reduction in the SCFA levels was correlated to the Lewy body formation in the enteric nervous system and sporadic PD. Although, different clinical histopathological investigations reported Lewy body formation in the enteric nervous system of PD patients,^109^ a direct correlation of enteric Lewy body formation due to a reduction in gut microbiome-derived SCFA is yet to be established. Rayman et al. recently proposed a mechanism for the early disease process through enteric Lewy body formation.^110^ Dysbiosis, together with a reduction in SFCA production, leads to an increase in gram-negative bacteria that produce LPS, curli protein (*E. coli*) and hydrogen sulfide production. These triggers facilitate damage to the intestinal barrier, initiate proinflammatory responses and mediate aSyn aggregation that may propagate from enteric neurons to the brainstem (Figure 8).

**Figure 9.**
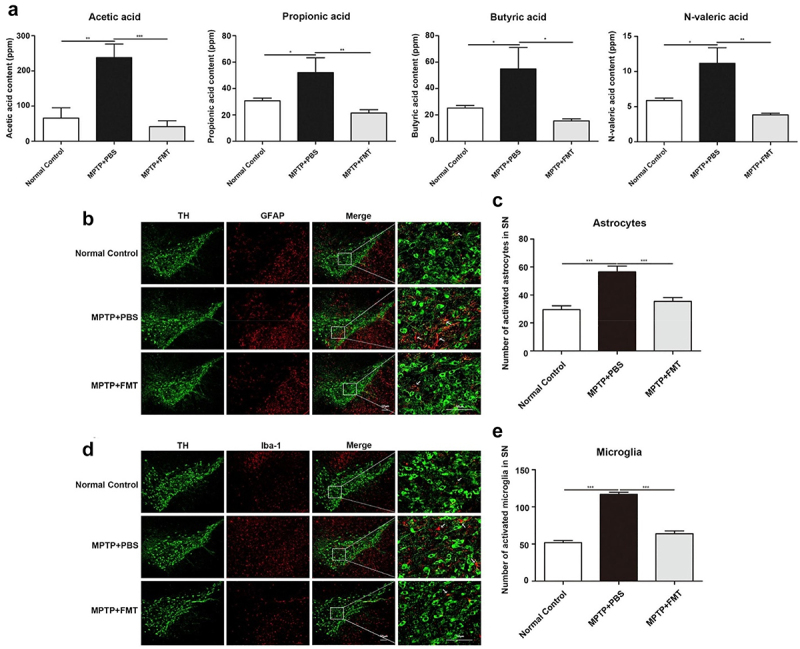
Increase in fecal SCFA associated with microglia-associated neuroinflammation. (a) Higher fecal SCFA content in mtpt-induced PD mice models. (b, c) higher astrocyte and (d, e) microglia activation and localization into substantia nigra. Scale bar (50 µm). Reprinted with permission from Elsevier [95].

Sun et al. discovered that gut microbiota from 1-methyl-4-phenyl-1,2,3,6-tetrahydropyridine (MPTP) induced PD mice models caused motor deficits and a decrease in striatal neurotransmitters, in comparison to healthy mice.^111^ Their 16S rRNA sequencing showed a decline in the *Firmicutes* phylum and *Clostridiales* order, while there was an increase in the *Proteobacteria* phylum, and *Turicibacterales* and *Enterobacteriales* orders in the fecal samples of PD mice, accompanied by elevated levels of fecal SCFA. Notably, fecal microbiota transplantation in PD mice led to a reduction in gut microbial imbalance, lowered fecal SCFA, improved motor function, and increased levels of striatal dopamine (DA) and 5-HT. Interestingly, the results of Sun et al. for PD are in agreement with Colombo et al. who also observed increased SCFA in AD mice models, as discussed above.^85^ Higher levels of SCFA were accompanied by microglial and astrocytic activation that can be attributed to SCFA-induced microglia-mediated neuroinflammation and neurodegeneration (Figure 9). Therefore, the multifaceted nature of SCFA in PD can be attributed to multiple pathways. SCFA can influence the immune system, potentially reducing neuroinflammation, a contributor to PD progression. By binding to GPCRs on immune cells, SCFA can attenuate the production of pro-inflammatory cytokines.^112^ SCFA can strengthen the BBB, potentially preventing harmful substances from entering the brain’s environment. A compromised BBB is a proposed risk factor for aSyn pathology.^113^ The gut microbiome, through metabolites like SCFA, plays a role in the synthesis of neurotransmitters such as dopamine, which is deficient in PD.^8^ Studies have indirectly suggested that SCFA might induce aSyn aggregation, however, it needs further investigation. Higher levels of SCFA can trigger astrocytes and microglia that localize to substantia nigra could promote neurodegeneration, as studied in MPTP-induced mice models.

### 5.3. Motor neuron disease

Motor neuron disease (MND) refers to a collection of degenerative neurological conditions that specifically target motor neurons, which are the cells responsible for regulating voluntary muscular movements, including speaking, walking, breathing, and swallowing. Amyotrophic Lateral Sclerosis (ALS), also referred to as Lou Gehrig’s illness, is the most prevalent type of MND. It is named after the renowned baseball player who was diagnosed with this condition.^114^ Motor neurons can be categorized into two types: upper motor neurons, which originate from the brain and travel to the spinal cord, and lower motor neurons, which originate in the spinal cord and directly connect with muscles. MND can impact both upper motor neurons and lower motor neurons. The gradual deterioration of motor neurons results in muscle weakness and atrophy, greatly hindering the patient’s capacity to regulate voluntary movements.^115^

#### 5.3.1. Gut microbiome-derived metabolites and MND

Emerging evidence suggests that the gut microbiome may play a role in MND pathogenesis, although this relationship is still being actively explored. Butyrate, for example, has neuroprotective effects, reducing neuroinflammation and oxidative stress, which are critical in protecting motor neurons. Similarly, elevated levels of neurotoxic metabolites such as quinolinic acid can contribute to motor neuron damage in ALS. TMAO’s role in promoting inflammatory pathways is particularly relevant in ALS, where inflammation is a key component of disease progression.^116^ Patients with MND often exhibit distinct microbial profiles compared to healthy individuals. Rowin et al. analyzed stool samples from five patients with ALS and MND, assessing for infection and indicators of intestinal inflammation.^117^ All five patients exhibited changes in their gut microbiome, notably a reduced diversity compared to healthy individuals with a relatively normal abundance. The microbiome predominantly consisted of bacteria from the Firmicutes and *Bacteroidetes* phyla. A low presence of *Ruminococcus* spp. was observed in three patients, which correlated with a decreased *Firmicutes/Bacteroidetes* (F/B) ratio. Furthermore, these patients showed evidence of intestinal inflammation and low SCFA levels.

Similar gut-dysbiosis and associated leaky gut epithelia were studied by Wu et al. in superoxide dismutase (SOD^G93A^) mice models.^118^ The mice presented decreased *Butyrivibrio Fibrisolvens*, *E. coli*, and *Fermicus* together with reduced levels of E-cadherin and ZO-1 in the intestinal epithelia. The antimicrobial peptide defensin 5α was also decreased and these changes led to increased systemic and intestinal levels of IL-17. McCombe et al. highlighted that the changes observed are linked with alterations in the microbiota composition, specifically noting decreased levels of *Butyrivibrio*, *Fibrisolvens*, *Fermicus*, and *E. coli*.^119^ Similar to AD and PD, SFCAs were concluded to be one of the key factors contributing to ALS neuropathology through microglia activation. Another gut microbiome-derived metabolite includes β-Methylamino-L-alanine (BMAA) which is produced by Cyanobacteria and Archaea. BMAA is a toxic amino acid that has structural similarity to L-serine, permeates across the gut-blood and BBB barriers and is found to be incorporated in different neuronal peptides in AD, PD, and ALS.^120^

Gut microbiome-derived metabolites modulate neuroinflammatory pathways, either promoting or mitigating inflammation in the CNS.^121^ Chronic neuroinflammation is a significant driver of motor neuron degeneration in MND. Metabolites like SCFAs and certain indoles possess antioxidant properties that help reduce oxidative stress, a key factor in motor neuron damage. These metabolites interact with the immune system, influencing both peripheral and central immune responses, which are crucial in the pathology of MND.

### 5.4. Huntington’s disease

Huntington’s disease (HD) is a genetic neurodegenerative condition marked by motor impairment, cognitive decline, and psychiatric issues; the gut microbiome has gained attention for its potential contributory role in the disease progression. The pathology of HD is primarily associated with an expanded CAG repeat in the huntingtin gene, but emerging research suggests that gut microbiome composition may influence disease manifestation and severity.^122^

#### 5.4.1. Gut microbiome-derived metabolites and HD

Studies have identified distinct microbial patterns in individuals with HD compared to healthy controls. For instance, there tends to be a notable decrease in overall microbial diversity.^123^ Certain microbial populations, such as those belonging to the phyla *Firmicutes* and *Bacteroidetes*, often exhibit altered abundances. Specifically, a decline in the abundance of butyrate-producing bacteria, which are pivotal for colonic health and systemic anti-inflammatory effects, has been observed.^124^ These associations suggest that microbial dysbiosis in HD may not only be a marker of the disease but could also participate in its pathophysiology by modulating peripheral inflammation and potentially influencing central neuroinflammatory processes.^125^

Du et al. conducted a study demonstrating gut dysbiosis, associated clinical manifestations and increased inflammatory cytokines of TNFα, ILs and interferons in 33 human patients with HD and 33 age-matched controls.^126^ They discovered that *Bilophila* were negatively correlated with proinflammatory cytokine IL-6, while a positive correlation was observed with *Intestinimonas* genus and anti-inflammatory cytokine IL-4. *Intestinimonas* was higher in HD patients which could be explained as a countermeasure to reduce inflammatory burden, however, future studies are required to further explain this observation.

Altered levels of gut-derived metabolites, such as SCFA, have also been implicated in HD. SCFA like butyrate is known for its role in maintaining gut barrier integrity and modulating immune responses, and reduced levels of butyrate-producing bacteria in HD patients might contribute to systemic inflammation.^127^ Moreover, metabolites such as quinolinic acid, a neurotoxin produced by certain gut bacteria, are found in elevated levels in the brains of HD patients and could be correlated to neurodegeneration.^128^ These metabolites can cross the BBB and directly affect neuronal health and function.^129^ Understanding how these metabolites contribute to the pathophysiology of HD could open new therapeutic avenues, such as microbiota-targeted therapies that aim to restore the balance of beneficial metabolites.

Tryptophan metabolites, particularly those produced through the Kyn pathway, have also been implicated in HD.^130^ These metabolites can permeate across BBB and influence CNS functions. Dysregulation of the Kyn pathway can lead to the production of neurotoxic metabolites such as quinolinic acid, which contributes to excitotoxicity and neuronal death, exacerbating HD symptoms. High TMAO levels are associated with increased oxidative stress and neuroinflammation, exacerbating neuronal damage and disease progression in HD.

### 5.5. Ataxia

Ataxia, a neurological sign characterized by impaired balance or coordination, can be caused by various conditions affecting the nervous system. This condition affects various parts of the nervous system, particularly the cerebellum, which is responsible for coordinating movement. The types of ataxias include cerebellar (linked to dysfunction or damage to the cerebellum), sensory (results from the loss of proprioception), and vestibular (caused by problems in the inner ear or the vestibular system).^131^

#### 5.5.1. Gut microbiome-derived metabolites and ataxia

Recent studies indicate a potential link between ataxia and alterations in the gut microbiome. Changes in the gut microbial composition may influence the central nervous system’s function through various pathways, including the modulation of systemic inflammation, the production of neuroactive metabolites, and the alteration of the gut-brain axis. Yu et al. explored the link between gut microbiota and Ataxia.^132^ They randomly selected 30 children with a history of intestinal surgery (HOIS) and 12 children without such a history (NHOIS) for their analysis. They identified significant differences in the abundance of certain microbial genera and phyla between the HOIS and NHOIS groups. This included five genera - *Acetivibrio, Catenibacterium, Comamonas, Paraeggerthella, and Rothia* - and the phylum *Candidatus Saccharibacteria*. In comparison to the HOIS group, the NHOIS group exhibited higher abundances of *Paraeggerthella*, *Rothia*, and *Candidatus Saccharibacteria*, while *Acetivibrio*, *Catenibacterium*, and *Comamonas* were less abundant. Between the NHOIS group and the control group, no significant differences were observed in any genus or phylum.

Specific microbial metabolites that play a role in the gut-brain axis and could be involved in the pathogenesis or progression of ataxia. For instance, dysbiosis in the gut microbiota might reduce the production of SCFA, which is known to exert neuroprotective effects through anti-inflammatory actions and serve as an energy source for colonic cells. Deficiencies in these SCFA may contribute to neuroinflammation and neurodegeneration associated with ataxic disorders.^133^ Furthermore, dysregulation of tryptophan metabolism by gut microbiota can affect the serotoninergic system and potentially the cerebellar function, thus influencing ataxic symptoms.^134^

Ongoing research is required to unravel the complexities of how gut microbiome-derived metabolites affect ataxic conditions, as these findings could pave the way for microbiota-targeted interventions in ataxia treatment.^16^

## 6. Conclusion

This review has comprehensively examined the complex association between the gut microbiota, the multifaceted role of their metabolites and pathologies of NDDs, emphasizing the intricate nature and importance of the gut-brain axis. The gut microbiome is composed of a wide range of species, which interact with the host’s physiology dynamically. This interaction has been extensively studied, with particular attention given to the variables that affect the diversity and abundance of these microbial species. The various roles of the microbiome have been examined, encompassing its contributions to metabolic processes, immune system functionality, integrity of gut epithelial and blood-brain barriers and its significant involvement in the creation of neurotransmitters. The interaction between the microbiome and pharmaceuticals, affecting drug efficacy and stability, further demonstrates the microbiome’s pervasive influence on human health.

The significance of microbial metabolites, such as SCFA, tryptophan metabolites, and secondary bile acids, has been illuminated, showcasing their roles in gut-brain signaling and overall health. In the context of NDDs, the review has delved into the alterations in the gut microbiome and its metabolites that may contribute to or exacerbate conditions such as AD, PD, MND, HD and Ataxia. Through the therapeutic implications chapter, we have reviewed the future potential of various interventions, from diet and pharmacological modulation to fecal microbiota transplantation and personalized medicine, each holding promises for modulating the gut microbiome to treat or prevent NDDs.

Despite these advances, challenges remain in studying the microbiome’s complexity, including technological limitations, standardization of methodologies, and the interpretation of vast datasets. However, the ongoing research and experimental tools within this domain are rapidly evolving, offering insights that are continuously refining our understanding of the microbiome.

## 7. Future directions

As we look to the future, research will likely focus on unraveling the causal relationships within the gut-brain axis and establishing more personalized therapeutic strategies. In particular, the cellular and animal models used to study the impact of gut-microbiome on neuropathologies, through the gut-brain axis need to consider the multifaceted nature of gut-microbes and metabolites. This will be required to match the results observed with human clinical observations and data obtained from biopsies and postmortem tissue samples. Advances in high-throughput sequencing, bioinformatics and integrative systems biology approaches are expected to drive this field forward, elucidating the nuanced interactions between microbial communities and the host. Furthermore, the potential for developing novel therapeutic techniques rooted in microbiome modulation is substantial. The potential interventions encompass a broad spectrum, spanning from prebiotics, probiotics, and synbiotics to more sophisticated methodologies, including genetic and microbial engineering. The use of longitudinal and interventional research will play a crucial role in substantiating the effectiveness of these treatments and comprehending the enduring consequences of microbiome manipulation.

Fecal Microbiota Transplantation (FMT) is a process where stool from a healthy donor is transferred into a patient’s gastrointestinal tract to restore a healthy, diverse microbiome.^135^ However, FMT carries risks such as introducing harmful organisms or genetic material, necessitating strict safety protocols.^136^ Ethical concerns also arise in donor selection and patient consent due to the microbiome’s complex impact on health. Regulatory bodies are working on guidelines to ensure FMT’s safety and effectiveness.^137^ Additionally, new drugs are being developed to target microbial metabolites, aiming to modulate inflammatory responses and oxidative stress in neurodegenerative diseases (NDDs). These drugs work by affecting the gut-brain axis, potentially altering disease progression in NDDs.^138^ Researchers are exploring ways to influence gut permeability, vagus nerve signaling, and the immune system’s interaction with the microbiome.^139^ Moreover, probiotics may offer neuroprotective effects by producing neurotransmitters – for example, certain *Lactobacillus* strains can generate GABA, and *Bifidobacteria* can produce acetylcholine.^8^ These neurotransmitters can directly or indirectly affect brain function.

There is increasing dissatisfaction with many of the classical conceptualizations of NDDs, including the role of Aβ in the course of dementia. Recent work shows Aβ to be an antimicrobial that may be excessively produced due to an aging-associated change in negative feedback.^140^ The impact of changes in the microbiome in NDDs may require a more detailed investigation of the cellular and systemic processes modulating the production and effects of inclusions such as Aβ, aSyn and TDP-43.

In summary, the gut microbiome constitutes a novel area of exploration in comprehending human biology, with significant promise for enhancing human well-being. Through further investigation of this intricate ecosystem, there is potential to unveil novel frameworks in the management of NDDs and other related fields, thereby facilitating the advent of a period characterized by enhanced individualized and efficacious healthcare.

## References

[1] Cryan JF, Dinan TG. Mind-altering microorganisms: the impact of the gut microbiota on brain and behaviour. Nat Rev Neurosci. 2012;13(10):701–30. doi:10.1038/nrn3346. 22968153

[2] Grenham S, Clarke G, Cryan JF, Dinan TG. Brain–gut–microbe communication in health and disease. Front Physiol. 2011;2:94. doi:10.3389/fphys.2011.00094. 22162969 PMC3232439

[3] Kowalski K, Mulak A. Brain-gut-microbiota axis in Alzheimer’s disease. J Neurogastroenterol Motil. 2019;25(1):48. doi:10.5056/jnm18087. 30646475 PMC6326209

[4] Houser MC, Tansey MG. The gut-brain axis: is intestinal inflammation a silent driver of Parkinson’s disease pathogenesis? NPJ Parkinson's Dis. 2017;3(1):3. doi:10.1038/s41531-016-0002-0. 28649603 PMC5445611

[5] Sherwin E, Sandhu KV, Dinan TG, Cryan JF. May the force be with you: the light and dark sides of the microbiota–gut–brain axis in neuropsychiatry. CNS Drugs. 2016;30(11):1019–1041. doi:10.1007/s40263-016-0370-3. 27417321 PMC5078156

[6] Ticinesi A, Tana C, Nouvenne A, Prati B, Lauretani F, Meschi T. Gut microbiota, cognitive frailty and dementia in older individuals: a systematic review. Clin Interv Aging. 2018;Volume 13:1497–1511. doi:10.2147/CIA.S139163. PMC6120508 30214170

[7] Erny D, de Angelis Al H, Jaitin D, Wieghofer P, Staszewski O, David E, Keren-Shaul H, Mahlakoiv T, Jakobshagen K, Buch T. Host microbiota constantly control maturation and function of microglia in the CNS. Nat Neurosci. 2015;18(7):965–977. doi:10.1038/nn.4030. 26030851 PMC5528863

[8] Strandwitz P. Neurotransmitter modulation by the gut microbiota. Brain Res. 2018;1693:128–133. doi:10.1016/j.brainres.2018.03.015. 29903615 PMC6005194

[9] Bourassa MW, Alim I, Bultman SJ, Ratan RR. Butyrate, neuroepigenetics and the gut microbiome: can a high fiber diet improve brain health? Neurosci Lett. 2016;625:56–63. doi:10.1016/j.neulet.2016.02.009. 26868600 PMC4903954

[10] Stilling RM, van de Wouw M, Clarke G, Stanton C, Dinan TG, Cryan JF. The neuropharmacology of butyrate: the bread and butter of the microbiota-gut-brain axis? Neurochem Int. 2016;99:110–132. doi:10.1016/j.neuint.2016.06.011. 27346602

[11] Agus A, Planchais J, Sokol H. Gut microbiota regulation of tryptophan metabolism in health and disease. Cell Host & Microbe. 2018;23(6):716–724. doi:10.1016/j.chom.2018.05.003. 29902437

[12] Kennedy PJ, Cryan JF, Dinan TG, Clarke G. Kynurenine pathway metabolism and the microbiota-gut-brain axis. Neuropharmacology. 2017;112:399–412. doi:10.1016/j.neuropharm.2016.07.002. 27392632

[13] Sarkar A, Lehto SM, Harty S, Dinan TG, Cryan JF, Burnet PWJ. Psychobiotics and the manipulation of bacteria–gut–brain signals. Trends Neurosci. 2016;39(11):763–781. doi:10.1016/j.tins.2016.09.002. 27793434 PMC5102282

[14] Dinan TG, Cryan JF. The microbiome-gut-brain axis in health and disease. Gastroenterol Clinics Of North Am. 2017;46(1):77–89. doi:10.1016/j.gtc.2016.09.007. 28164854

[15] Javed I, Cui X, Wang X, Mortimer M, Andrikopoulos N, Li Y, Davis TP, Zhao Y, Ke PC, Chen C. Implications of the human gut–brain and gut–cancer axes for future nanomedicine. ACS Nano. 2020;14(11):14391–14416. doi:10.1021/acsnano.0c07258. 33138351

[16] Morais LH, Schreiber IH, Mazmanian SK. The gut microbiota–brain axis in behaviour and brain disorders. Nat Rev Microbiol. 2021;19(4):241–255. doi:10.1038/s41579-020-00460-0. 33093662

[17] Forsythe P, Bienenstock J, Kunze WA. Vagal pathways for microbiome-brain-gut axis communication. Microb Endocrinol: The Microbiota-Gut-Brain Axis In Health And Disease. 2014;817:115–133. doi:10.1007/978-1-4939-0897-4_5. 24997031

[18] Breit S, Kupferberg A, Rogler G, Hasler G. Vagus nerve as modulator of the brain–gut axis in psychiatric and inflammatory disorders. Front Psychiatry. 2018;9:44. doi:10.3389/fpsyt.2018.00044. 29593576 PMC5859128

[19] Tanida M, Yamano T, Maeda K, Okumura N, Fukushima Y, Nagai K. Effects of intraduodenal injection of lactobacillus johnsonii La1 on renal sympathetic nerve activity and blood pressure in urethane-anesthetized rats. Neurosci Lett. 2005;389(2):109–114. doi:10.1016/j.neulet.2005.07.036. 16118039

[20] Carabotti M, Scirocco A, Maselli MA, Severi C. The gut-brain axis: interactions between enteric microbiota, central and enteric nervous systems. Ann Of Gastroenterol: Q Publ Of The Hellenic Soc Of Gastroenterol. 2015;28:203. PMC4367209 25830558

[21] Mayer EA, Tillisch K, Gupta A. Gut/Brain axis and the microbiota. J Clin Invest. 2015;125(3):926–938. doi:10.1172/JCI76304. 25689247 PMC4362231

[22] Pavlov VA, Tracey KJ. The vagus nerve and the inflammatory reflex—linking immunity and metabolism. Nat Rev Endocrinol. 2012;8(12):743–754. doi:10.1038/nrendo.2012.189. 23169440 PMC4082307

[23] Bonaz B, Picq C, Sinniger V, Mayol J-F, Clarençon D. Vagus nerve stimulation: from epilepsy to the cholinergic anti‐inflammatory pathway. Neurogastroenterology & Motil. 2013;25(3):208–221. doi:10.1111/nmo.12076. 23360102

[24] Braak H, Rüb U, Gai WP, Del Tredici K. Idiopathic Parkinson’s disease: possible routes by which vulnerable neuronal types may be subject to neuroinvasion by an unknown pathogen. J Neural Transm. 2003;110:517–536. doi:10.1007/s00702-002-0808-2. 12721813

[25] Kim S, Kwon S-H, Kam T-I, Panicker N, Karuppagounder SS, Lee S, Lee JH, Kim WR, Kook M, Foss CA. Transneuronal propagation of pathologic α-synuclein from the gut to the brain models Parkinson’s disease. Neuron. 2019;103(4):627–641.e7. doi:10.1016/j.neuron.2019.05.035. 31255487 PMC6706297

[26] Siopi E, Galerne M, Rivagorda M, Saha S, Moigneu C, Moriceau S, Bigot M, Oury F, Lledo P-M. Gut microbiota changes require vagus nerve integrity to promote depressive-like behaviors in mice. Mol Psychiatry. 2023;28:1–11. doi:10.1038/s41380-023-02071-6. 37131071 PMC10615761

[27] Yano JM, Yu K, Donaldson GP, Shastri GG, Ann P, Ma L, Nagler CR, Ismagilov RF, Mazmanian SK, Hsiao EY. Indigenous bacteria from the gut microbiota regulate host serotonin biosynthesis. Cell. 2015;161(2):264–276. doi:10.1016/j.cell.2015.02.047. 25860609 PMC4393509

[28] Margolis KG, Gershon MD. Enteric neuronal regulation of intestinal inflammation. Trends Neurosci. 2016;39(9):614–624. doi:10.1016/j.tins.2016.06.007. 27450201 PMC5002370

[29] Braak H, de Vos Rai, Bohl J, Del Tredici K, de Vos RAI. Gastric α-synuclein immunoreactive inclusions in Meissner’s and Auerbach’s plexuses in cases staged for Parkinson’s disease-related brain pathology. Neurosci Lett. 2006;396(1):67–72. doi:10.1016/j.neulet.2005.11.012. 16330147

[30] Latorre R, Sternini C, De Giorgio R, Greenwood‐Van Meerveld B. Enteroendocrine cells: a review of their role in brain–gut communication. Neurogastroenterology & Motil. 2016;28(5):620–630. doi:10.1111/nmo.12754. PMC4842178 26691223

[31] Rinninella E, Raoul P, Cintoni M, Franceschi F, Miggiano GAD, Gasbarrini A, Mele MC. What is the healthy gut microbiota composition? A changing ecosystem across age, environment, diet, and diseases. Microorganisms. 2019;7(1):14. doi:10.3390/microorganisms7010014. 30634578 PMC6351938

[32] Qin J, Li R, Raes J, Arumugam M, Burgdorf KS, Manichanh C, Nielsen T, Pons N, Levenez F, Yamada T. A human gut microbial gene catalogue established by metagenomic sequencing. Nature. 2010;464(7285):59–65. doi:10.1038/nature08821. 20203603 PMC3779803

[33] Dominguez-Bello MG, Costello EK, Contreras M, Magris M, Hidalgo G, Fierer N, Knight R. Delivery mode shapes the acquisition and structure of the initial microbiota across multiple body habitats in newborns. Proc Natl Acad Sci USA; Vol. 107. 2010. p. 11971–11975. doi:10.1073/pnas.1002601107. PMC2900693 20566857

[34] Zheng J, Yuan X, Cheng G, Jiao S, Feng C, Zhao X, Yin H, Du Y, Liu H. Chitosan oligosaccharides improve the disturbance in glucose metabolism and reverse the dysbiosis of gut microbiota in diabetic mice. Carbohydr Polym. 2018;190:77–86. doi:10.1016/j.carbpol.2018.02.058. 29628262

[35] Fernandez-Julia PJ, Munoz-Munoz J, van Sinderen D. A comprehensive review on the impact of β-glucan metabolism by Bacteroides and bifidobacterium species as members of the gut microbiota. Int J Biol Macromol. 2021;181:877–889. doi:10.1016/j.ijbiomac.2021.04.069. 33864864

[36] El Rabey HA, Almutairi FM, Alalawy AI, Ma A-D, Sakran MI, Zidan NS, Tayel AA. Augmented control of drug-resistant Candida spp. Via fluconazole loading into fungal chitosan nanoparticles. Int J Biol Macromol. 2019;141:511–516. doi:10.1016/j.ijbiomac.2019.09.036. 31499111

[37] Satokari R. High intake of sugar and the balance between pro-and anti-inflammatory gut bacteria. Nutrients2020. 2020;12(5):1348. doi:10.3390/nu12051348. PMC7284805 32397233

[38] Li H-L, Lu L, Wang X-S, Qin L-Y, Wang P, Qiu S-P, Wu H, Huang F, Zhang B-B, Shi H-L. Alteration of gut microbiota and inflammatory cytokine/chemokine profiles in 5-fluorouracil induced intestinal mucositis. Front Cell Infect Microbiol. 2017;7:455. doi:10.3389/fcimb.2017.00455. 29124041 PMC5662589

[39] van der Hee B, Wells JM. Microbial regulation of host physiology by short-chain fatty acids. Trends Microbiol. 2021;29(8):700–712. doi:10.1016/j.tim.2021.02.001. 33674141

[40] Rios-Covian D, González S, Nogacka AM, Arboleya S, Salazar N, Gueimonde M, de Los Reyes-Gavilán Cg, de Los Reyes-Gavilán CG. An overview on fecal branched short-chain fatty acids along human life and as related with body mass index: associated dietary and anthropometric factors. Front Microbiol. 2020;11:973. doi:10.3389/fmicb.2020.00973. 32547507 PMC7271748

[41] Zhu X, Li B, Lou P, Dai T, Chen Y, Zhuge A, Yuan Y, Li L. The relationship between the gut microbiome and neurodegenerative diseases. Neurosci Bull. 2021;37(10):1510–1522. doi:10.1007/s12264-021-00730-8. 34216356 PMC8490573

[42] Sharma VK, Singh TG, Prabhakar NK, Mannan A. Kynurenine metabolism and alzheimer’s disease: the potential targets and approaches. Neurochem Res. 2022;47(6):1459–1476. doi:10.1007/s11064-022-03546-8. 35133568

[43] Sheibani M, Shayan M, Khalilzadeh M, Soltani ZE, Jafari-Sabet M, Ghasemi M, Dehpour AR. Kynurenine pathway and its role in neurologic, psychiatric, and inflammatory bowel diseases. Mol Biol Rep. 2023;50(12):10409–10425. doi:10.1007/s11033-023-08859-7. 37848760

[44] Chen P, Geng X. Research progress on the kynurenine pathway in the prevention and treatment of Parkinson’s disease. J Enzym Inhib Med Chem. 2023;38(1):2225800. doi:10.1080/14756366.2023.2225800. PMC10312032 37381707

[45] Wahlström A, Sayin SI, Marschall H-U, Bäckhed F. Intestinal crosstalk between bile acids and microbiota and its impact on host metabolism. Cell Metab. 2016;24(1):41–50. doi:10.1016/j.cmet.2016.05.005. 27320064

[46] Jia W, Xie G, Jia W. Bile acid–microbiota crosstalk in gastrointestinal inflammation and carcinogenesis. Nat Rev Gastroenterol Hepatol. 2018;15(2):111–128. doi:10.1038/nrgastro.2017.119. 29018272 PMC5899973

[47] Patterson E, Cryan JF, Fitzgerald GF, Ross RP, Dinan TG, Stanton C. Gut microbiota, the pharmabiotics they produce and host health. Proceedings of the Nutrition Society; Vol. 73. 2014. p. 477–489. doi:10.1017/S0029665114001426. 25196939

[48] Hurley MJ, Bates R, Macnaughtan J, V SA. Bile acids and neurological disease. Pharmacol Ther. 2022;240:108311. doi:10.1016/j.pharmthera.2022.108311. 36400238

[49] Nie K, Li Y, Zhang J, Gao Y, Qiu Y, Gan R, Zhang Y, Wang L. Distinct bile acid signature in Parkinson’s disease with mild cognitive impairment. Front Neurol. 2022;13:897867. doi:10.3389/fneur.2022.897867. 35860484 PMC9289438

[50] Wang S, Xu C, Liu H, Wei W, Zhou X, Qian H, Zhou L, Zhang H, Wu L, Zhu C. Connecting the gut microbiota and neurodegenerative diseases: the role of bile acids. Mol Neurobiol. 2023;60(8):4618–4640. doi:10.1007/s12035-023-03340-9. 37121952

[51] Zhang Y, Yu W, Zhang L, Wang M, Chang W. The interaction of polyphenols and the gut microbiota in neurodegenerative diseases. Nutrients. 2022;14(24):5373. doi:10.3390/nu14245373. 36558531 PMC9785743

[52] Marino A, Battaglini M, Moles N, Ciofani G. Natural antioxidant compounds as potential pharmaceutical tools against neurodegenerative diseases. ACS Omega. 2022;7(30):25974–25990. doi:10.1021/acsomega.2c03291. 35936442 PMC9352343

[53] Vicente-Zurdo D, Gómez-Mejía E, Rosales-Conrado N, León-González ME. A comprehensive analytical review of polyphenols: evaluating neuroprotection in Alzheimer’s disease. Int J Mol Sci. 2024;25(11):5906. doi:10.3390/ijms25115906. 38892094 PMC11173253

[54] Li D, Luo F, Guo T, Han S, Wang H, Lin Q. Targeting nf-κB pathway by dietary lignans in inflammation: expanding roles of gut microbiota and metabolites. Crit Rev Food Sci Nutr. 2023;63(22):5967–5983. doi:10.1080/10408398.2022.2026871. 35068283

[55] Praveenraj SS, Sonali S, Anand N, Tousif HA, Vichitra C, Kalyan M, Kanna PV, Chandana KA, Shasthara P, Mahalakshmi AM. The role of a gut microbial-derived metabolite, trimethylamine N-oxide (TMAO), in neurological disorders. Mol Neurobiol. 2022;59(11):6684–6700. doi:10.1007/s12035-022-02990-5. 35986843

[56] Botchway BOA, Okoye FC, Chen Y, Arthur WE, Fang M. Alzheimer disease: recent updates on apolipoprotein E and gut microbiome mediation of oxidative stress, and prospective interventional agents. Aging Dis. 2022;13(1):87. doi:10.14336/AD.2021.0616. 35111364 PMC8782546

[57] Liang J, Wang Y, Liu B, Dong X, Cai W, Zhang N, Zhang H. Deciphering the intricate linkage between the gut microbiota and Alzheimer’s disease: elucidating mechanistic pathways promising therapeutic strategies. CNS Neurosci Ther. 2024;30:e14704. doi:10.1111/cns.14704. 38584341 PMC10999574

[58] Wang Y. The role of the gut microbiota and microbial metabolites in the pathogenesis of alzheimer’s disease. CNS & Neurological Disord-Drug Targets (Former Curr Drug Targets-CNS & Neurological Disord). 2023;22(4):577–598. doi:10.2174/1871527321666220417005115. 35440320

[59] Shome A, Chawla V, Chawla PA, Chawla PA. Neuroprotective effect of natural indole and β-carboline alkaloids against Parkinson’s disease: an overview. Curr Med Chem. 2024;31(38):6251–6271. doi:10.2174/0929867331666230913100624. 37702172

[60] Konopelski P, Mogilnicka I. Biological effects of indole-3-propionic acid, a gut microbiota-derived metabolite, and its precursor tryptophan in mammals’ health and disease. Int J Mol Sci. 2022;23(3):1222. doi:10.3390/ijms23031222. 35163143 PMC8835432

[61] Zhou Y, Chen Y, He H, Peng M, Zeng M, Sun H. The role of the indoles in microbiota-gut-brain axis and potential therapeutic targets: a focus on human neurological and neuropsychiatric diseases. Neuropharmacology. 2023;239:109690. doi:10.1016/j.neuropharm.2023.109690. 37619773

[62] Pasha AR, Khan A, Ullah S, Halim SA, Alharthy RD, Anwar MU, Hussain J, Naseer MM, Kashtoh H, Al-Harrasi A. Indole-based thiosemicarbazones for neurodegenerative diseases as prolyl oligopeptidase inhibitors. J Mol Struct. 2024;1312:138666. doi:10.1016/j.molstruc.2024.138666.

[63] Golan N, Engelberg Y, Landau M. Structural mimicry in microbial and antimicrobial amyloids. Annu Rev Biochem. 2022;91(1):403–422. doi:10.1146/annurev-biochem-032620-105157. 35729071

[64] Elkins M, Jain N, Ç T. The menace within: bacterial amyloids as a trigger for autoimmune and neurodegenerative diseases. Curr Opin Microbiol. 2024;79:102473. doi:10.1016/j.mib.2024.102473. 38608623 PMC11162901

[65] Javed I, Zhang Z, Adamcik J, Andrikopoulos N, Li Y, Otzen DE, Lin S, Mezzenga R, Davis TP, Ding F. Accelerated amyloid beta pathogenesis by bacterial amyloid FapC. Adv Sci. 2020;7(18):2001299. doi:10.1002/advs.202001299. PMC7509637 32999841

[66] Sampson TR, Challis C, Jain N, Moiseyenko A, Ladinsky MS, Shastri GG, Thron T, Needham BD, Horvath I, Debelius JW. A gut bacterial amyloid promotes α-synuclein aggregation and motor impairment in mice. Elife. 2020;9:e53111. doi:10.7554/eLife.53111. 32043464 PMC7012599

[67] Fernández-Calvet A, Matilla-Cuenca L, Izco M, Navarro S, Serrano M, Ventura S, Blesa J, Herráiz M, Alkorta-Aranburu G, Galera S. Gut microbiota produces biofilm-associated amyloids with potential for neurodegeneration. Nat Commun. 2024;15(1):4150. doi:10.1038/s41467-024-48309-x. 38755164 PMC11099085

[68] Ali SA, Chung KHK, Forgham H, Olsen WP, Kakinen A, Balaji A, Otzen DE, Davis TP, Javed I. Alzheimer’s progenitor amyloid-β targets and dissolves microbial amyloids and impairs biofilm function. Adv Sci. 2023;10(29):2301423. doi:10.1002/advs.202301423. PMC10582422 37594661

[69] Ke PC, Sani M-A, Ding F, Kakinen A, Javed I, Separovic F, Davis TP, Mezzenga R. Implications of peptide assemblies in amyloid diseases. Chem Soc Rev. 2017;46(21):6492–6531. doi:10.1039/C7CS00372B. 28702523 PMC5902192

[70] Dodiya HB, Frith M, Sidebottom A, Cao Y, Koval J, Chang E, Sisodia SS. Synergistic depletion of gut microbial consortia, but not individual antibiotics, reduces amyloidosis in APPPS1-21 Alzheimer’s transgenic mice. Sci Rep. 2020;10(1):8183. doi:10.1038/s41598-020-64797-5. 32424118 PMC7235236

[71] Cerovic M, Forloni G, Balducci C. Neuroinflammation and the gut microbiota: possible alternative therapeutic targets to counteract Alzheimer’s disease? Front Aging Neurosci. 2019;11:284. doi:10.3389/fnagi.2019.00284. 31680937 PMC6813195

[72] Cattaneo A, Cattane N, Galluzzi S, Provasi S, Lopizzo N, Festari C, Ferrari C, Guerra UP, Paghera B, Muscio C. Association of brain amyloidosis with pro-inflammatory gut bacterial taxa and peripheral inflammation markers in cognitively impaired elderly. Neurobiol Aging. 2017;49:60–68. doi:10.1016/j.neurobiolaging.2016.08.019. 27776263

[73] Jiang C, Li G, Huang P, Liu Z, Zhao B. The gut microbiota and Alzheimer’s disease. J Alzheimer’s Disease. 2017;58(1):1–15. doi:10.3233/JAD-161141. 28372330

[74] Pistollato F, Sumalla Cano S, Elio I, Masias Vergara M, Giampieri F, Battino M. Role of gut microbiota and nutrients in amyloid formation and pathogenesis of Alzheimer disease. Nutr Rev. 2016;74(10):624–634. doi:10.1093/nutrit/nuw023. 27634977

[75] Zhao Y, Lukiw WJ. Microbiome-generated amyloid and potential impact on amyloidogenesis in Alzheimer’s disease (AD). J Nat Sci. 2015;7:1. PMC4469284 26097896

[76] Leblhuber F, Steiner K, Schuetz B, Fuchs D, Gostner JM. Probiotic supplementation in patients with Alzheimer’s dementia-an explorative intervention study. Curr Alzheimer Res. 2018;15(12):1106–1113. doi:10.2174/1389200219666180813144834. 30101706 PMC6340155

[77] Vogt NM, Kerby RL, Ka D-M, Harding SJ, Merluzzi AP, Johnson SC, Carlsson CM, Asthana S, Zetterberg H, Blennow K. Gut microbiome alterations in Alzheimer’s disease. Sci Rep. 2017;7(1):13537. doi:10.1038/s41598-017-13601-y. 29051531 PMC5648830

[78] Nho K, Kueider-Paisley A, MahmoudianDehkordi S, Arnold M, Risacher SL, Louie G, Blach C, Baillie R, Han X, Kastenmüller G. Altered bile acid profile in mild cognitive impairment and Alzheimer’s disease: relationship to neuroimaging and CSF biomarkers. Alzheimer’s & Dementia. 2019;15:232–244. doi:10.1016/j.jalz.2018.08.012. PMC6454538 30337152

[79] Mintun MA, Lo AC, Duggan Evans C, Wessels AM, Ardayfio PA, Andersen SW, Shcherbinin S, Sparks J, Sims JR, Brys M. Donanemab in early Alzheimer’s disease. N Engl J Med. 2021;384(18):1691–1704. doi:10.1056/NEJMoa2100708. 33720637

[80] Cao T, Zhou X, Zheng X, Cui Y, Tsien JZ, Li C, Wang H. Histone deacetylase inhibitor alleviates the neurodegenerative phenotypes and histone dysregulation in presenilins-deficient mice. Front Aging Neurosci. 2018;10:137. doi:10.3389/fnagi.2018.00137. 29867447 PMC5962686

[81] Govindarajan N, Agis-Balboa RC, Walter J, Sananbenesi F, Fischer A. Sodium butyrate improves memory function in an Alzheimer’s disease mouse model when administered at an advanced stage of disease progression. J Alzheimer’s Disease. 2011;26:187–197. doi:10.3233/JAD-2011-110080. 21593570

[82] Fernando W, Martins IJ, Morici M, Bharadwaj P, Rainey-Smith SR, Lim WLF, Martins RN. Sodium butyrate reduces brain amyloid-β levels and improves cognitive memory performance in an Alzheimer’s disease transgenic mouse model at an early disease stage. J Alzheimer’s Disease. 2020;74(1):91–99. doi:10.3233/JAD-190120. 31958090

[83] Ho L, Ono K, Tsuji M, Mazzola P, Singh R, Pasinetti GM. Protective roles of intestinal microbiota derived short chain fatty acids in Alzheimer’s disease-type beta-amyloid neuropathological mechanisms. Expert Rev Neurother. 2018;18(1):83–90. doi:10.1080/14737175.2018.1400909. 29095058 PMC5958896

[84] Huang H-J, Chen J-L, Liao J-F, Chen Y-H, Chieu M-W, Ke Y-Y, Hsu C-C, Tsai Y-C, Hsieh-Li HM. Lactobacillus plantarum PS128 prevents cognitive dysfunction in Alzheimer’s disease mice by modulating propionic acid levels, glycogen synthase kinase 3 beta activity, and gliosis. BMC Complement Med Ther. 2021;21(1):1–16. doi:10.1186/s12906-021-03426-8. 34627204 PMC8502419

[85] Colombo AV, Sadler RK, Llovera G, Singh V, Roth S, Heindl S, Sebastian Monasor L, Verhoeven A, Peters F, Parhizkar S. Microbiota-derived short chain fatty acids modulate microglia and promote Aβ plaque deposition. Elife. 2021;10:e59826. doi:10.7554/eLife.59826. 33845942 PMC8043748

[86] Zhang L, Wang Y, Xiayu X, Shi C, Chen W, Song N, Fu X, Zhou R, Y-F X, Huang L. Altered gut microbiota in a mouse model of Alzheimer’s disease. J Alzheimer’s Disease. 2017;60(4):1241–1257. doi:10.3233/JAD-170020. 29036812

[87] Zheng J, Zheng S-J, Cai W-J, Yu L, Yuan B-F, Feng Y-Q. Stable isotope labeling combined with liquid chromatography-tandem mass spectrometry for comprehensive analysis of short-chain fatty acids. Anal Chim Acta. 2019;1070:51–59. doi:10.1016/j.aca.2019.04.021. 31103167

[88] Syeda T, Sanchez-Tapia M, Pinedo-Vargas L, Granados O, Cuervo-Zanatta D, Rojas-Santiago E, Díaz-Cintra S, Torres N, Perez-Cruz C, Albensi B. Bioactive food abates metabolic and synaptic alterations by modulation of gut microbiota in a mouse model of Alzheimer’s disease. J Alzheimer’s Disease. 2018;66(4):1657–1682. doi:10.3233/JAD-180556. 30475761

[89] Kong Y, Jiang B, Luo X. Gut microbiota influences Alzheimer’s disease pathogenesis by regulating acetate in drosophila model. Future Microbiol. 2018;13(10):1117–1128. doi:10.2217/fmb-2018-0185. 30043649

[90] Wang X, Sun G, Feng T, Zhang J, Huang X, Wang T, Xie Z, Chu X, Yang J, Wang H. Sodium oligomannate therapeutically remodels gut microbiota and suppresses gut bacterial amino acids-shaped neuroinflammation to inhibit Alzheimer’s disease progression. Cell Res. 2019;29(10):787–803. doi:10.1038/s41422-019-0216-x. 31488882 PMC6796854

[91] Yilmaz A, Geddes T, Han B, Bahado-Singh RO, Wilson GD, Imam K, Maddens M, Graham SF. Diagnostic biomarkers of Alzheimer’s disease as identified in saliva using 1H nmr-based metabolomics. J Alzheimer’s Disease. 2017;58(2):355–359. doi:10.3233/JAD-161226. 28453477

[92] Figueira J, Jonsson P, Adolfsson AN, Adolfsson R, Nyberg L, A Ö. NMR analysis of the human saliva metabolome distinguishes dementia patients from matched controls. Mol Biosyst. 2016;12(8):2562–2571. doi:10.1039/C6MB00233A. 27265744

[93] Cui M, Jiang Y, Zhao Q, Zhu Z, Liang X, Zhang K, Wu W, Dong Q, An Y, Tang H. Metabolomics and incident dementia in older Chinese adults: the shanghai aging study. Alzheimer’s & Dementia. 2020;16:779–788. doi:10.1002/alz.12074. 32270572

[94] Wu L, Han Y, Zheng Z, Peng G, Liu P, Yue S, Zhu S, Chen J, Lv H, Shao L. Altered gut microbial metabolites in amnestic mild cognitive impairment and Alzheimer’s disease: signals in host–microbe interplay. Nutrients. 2021;13(1):228. doi:10.3390/nu13010228. 33466861 PMC7829997

[95] Hufnagel DA, Ç T, Chapman MR. Disease to dirt: the biology of microbial amyloids. PloS Pathog. 2013;9(11):e1003740. doi:10.1371/journal.ppat.1003740. 24278013 PMC3836715

[96] Zhao Y, Dua P, Lukiw WJ. Microbial sources of amyloid and relevance to amyloidogenesis and Alzheimer’s disease (AD). J Alzheimers Dis Parkinsonism. 2015;5:177. doi:10.4172/2161-0460.1000177. 25977840 PMC4428612

[97] Koppel K, Tang H, Javed I, Parsa M, Mortimer M, Davis TP, Lin S, Chaffee AL, Ding F, Ke PC. Elevated amyloidoses of human IAPP and amyloid beta by lipopolysaccharide and their mitigation by carbon quantum dots. Nanoscale. 2020;12(23):12317–12328. doi:10.1039/D0NR02710C. 32490863 PMC7325865

[98] Er Al D, Constantinescu R, Thompson JP, Biglan KM, Holloway RG, Kieburtz K, Marshall FJ, Ravina BM, Schifitto G, Siderowf A. Projected number of people with Parkinson disease in the most populous nations, 2005 through 2030. Neurology. 2007;68(5):384–386. doi:10.1212/01.wnl.0000247740.47667.03. 17082464

[99] Wirdefeldt K, Adami H-O, Cole P, Trichopoulos D, Mandel J. Epidemiology and etiology of Parkinson’s disease: a review of the evidence. Eur J Epidemiol. 2011;26(S1):1–58. doi:10.1007/s10654-011-9581-6. 21626386

[100] Svensson E, Horváth‐Puhó E, Thomsen RW, Djurhuus JC, Pedersen L, Borghammer P, Sørensen HT. Vagotomy and subsequent risk of P arkinson’s disease. Ann Neurol. 2015;78(4):522–529. doi:10.1002/ana.24448. 26031848

[101] Hill‐Burns EM, Debelius JW, Morton JT, Wissemann WT, Lewis MR, Wallen ZD, Peddada SD, Factor SA, Molho E, Zabetian CP. Parkinson’s disease and Parkinson’s disease medications have distinct signatures of the gut microbiome. Mov Disord. 2017;32:739–749. doi:10.1002/mds.26942. 28195358 PMC5469442

[102] Jager WADH, Bethlem J. The distribution of Lewy bodies in the central and autonomic nervous systems in idiopathic paralysis agitans. J Neurol Neurosurg Psychiatry. 1960;23(4):283. doi:10.1136/jnnp.23.4.283. 13711997 PMC497426

[103] Lee WY, Yoon WT, Shin HY, Jeon SH, Rhee P. Helicobacter pylori infection and motor fluctuations in patients with Parkinson’s disease. Mov Disord. 2008;23:1696–1700. doi:10.1002/mds.22190. 18649391

[104] Weller C, Charlett A, Oxlade NL, Dobbs SM, Dobbs RJ, Peterson DW, Bjarnason IT. Role of chronic infection and inflammation in the gastrointestinal tract in the etiology and pathogenesis of idiopathic parkinsonism: part 3: predicted probability and gradients of severity of idiopathic parkinsonism based on H. pylori antibody profile. Helicobacter. 2005;10(4):288–297. doi:10.1111/j.1523-5378.2005.00329.x. 16104944

[105] Dobbs RJ, Dobbs SM, Weller C, Bjarnason IT, Oxlade NL, Charlett A, Ma A, Kerwin RW, Mahler RF, Price AB. Role of chronic infection and inflammation in the gastrointestinal tract in the etiology and pathogenesis of idiopathic parkinsonism: part 1: eradication of Helicobacter in the cachexia of idiopathic parkinsonism. Helicobacter. 2005;10(4):267–275. doi:10.1111/j.1523-5378.2005.00331.x. 16104942

[106] Nyholm D, Hellström PM. Effects of Helicobacter pylori on levodopa pharmacokinetics. J Parkinsons Dis. 2021;11(1):61–69. doi:10.3233/JPD-202298. 33164946 PMC7990449

[107] Mridula KR, Borgohain R, Chandrasekhar Reddy V, Bandaru VCS, Suryaprabha T. Association of Helicobacter pylori with Parkinson’s disease. J Clin Neurol. 2017;13:181–186. doi:10.3988/jcn.2017.13.2.181. 28406585 PMC5392461

[108] Unger MM, Spiegel J, Dillmann K-U, Grundmann D, Philippeit H, Bürmann J, K F, Schwiertz A, Schäfer K-H. Short chain fatty acids and gut microbiota differ between patients with Parkinson’s disease and age-matched controls. Parkinsonism Relat Disord. 2016;32:66–72. doi:10.1016/j.parkreldis.2016.08.019. 27591074

[109] Rao M, Gershon MD. The bowel and beyond: the enteric nervous system in neurological disorders. Nat Rev Gastroenterol Hepatol. 2016;13(9):517–528. doi:10.1038/nrgastro.2016.107. 27435372 PMC5005185

[110] Ryman S, Vakhtin AA, Richardson SP, Lin HC. Microbiome–gut–brain dysfunction in prodromal and symptomatic Lewy body diseases. J Neurol. 2023;270(2):746–758. doi:10.1007/s00415-022-11461-9. 36355185 PMC9886597

[111] Sun M-F, Zhu Y-L, Zhou Z-L, Jia X-B, Xu Y-D, Yang Q, Cui C, Shen Y-Q. Neuroprotective effects of fecal microbiota transplantation on mptp-induced Parkinson’s disease mice: gut microbiota, glial reaction and TLR4/TNF-α signaling pathway. Brain Behav Immun. 2018;70:48–60. doi:10.1016/j.bbi.2018.02.005. 29471030

[112] Vinolo MAR, Rodrigues HG, Nachbar RT, Curi R. Regulation of inflammation by short chain fatty acids. Nutrients. 2011;3(10):858–876. doi:10.3390/nu3100858. 22254083 PMC3257741

[113] Braniste V, Al-Asmakh M, Kowal C, Anuar F, Abbaspour A, Tóth M, Korecka A, Bakocevic N, Ng LG, Kundu P. The gut microbiota influences blood-brain barrier permeability in mice. Sci Transl Med. 2014;6(263):ra263158–ra263158. doi:10.1126/scitranslmed.3009759. PMC4396848 25411471

[114] Abati E, Manini A, Comi G, Corti, Pietro S, Corti S. Inhibition of myostatin and related signaling pathways for the treatment of muscle atrophy in motor neuron diseases. Cell Mol Life Sci. 2022;79(7):374. doi:10.1007/s00018-022-04408-w. 35727341 PMC9213329

[115] Forshew D A, Bromberg M B. A survey of clinicians’ practice in the symptomatic treatment of ALS. Amyotroph Lateral Scler And Other Mot Neuron Disord. 2003;4:258–263. doi:10.1080/14660820310017344. 14753660

[116] Ma Y-Y, Li X, Yu J-T, Wang Y-J. Therapeutics for neurodegenerative diseases by targeting the gut microbiome: from bench to bedside. Transl Neurodegener. 2024;13(1):12. doi:10.1186/s40035-024-00404-1. 38414054 PMC10898075

[117] Rowin J, Xia Y, Jung B, Sun J. Gut inflammation and dysbiosis in human motor neuron disease. Physiol Rep. 2017;5(18):e13443. doi:10.14814/phy2.13443. 28947596 PMC5617930

[118] Wu S, Yi J, Zhang Y, Zhou J, Sun J. Leaky intestine and impaired microbiome in an amyotrophic lateral sclerosis mouse model. Physiol Rep. 2015;3(4):e12356. doi:10.14814/phy2.12356. 25847918 PMC4425962

[119] McCombe PA, Henderson RD, Lee A, Lee JD, Woodruff TM, Restuadi R, McRae A, Wray NR, Ngo S, Steyn FJ. Gut microbiota in ALS: possible role in pathogenesis? Expert Rev Neurother. 2019;19(9):785–805. doi:10.1080/14737175.2019.1623026. 31122082

[120] Brenner SR. Blue-green algae or cyanobacteria in the intestinal micro-flora may produce neurotoxins such as beta-N-Methylamino-L-Alanine (BMAA) which may be related to development of amyotrophic lateral sclerosis, Alzheimer’s disease and Parkinson-dementia-complex in humans and equine motor neuron disease in horses. Med Hypotheses. 2013;80:103. doi:10.1016/j.mehy.2012.10.010. 23146671

[121] Borbolis F, Mytilinaiou E, Palikaras K. The crosstalk between microbiome and mitochondrial homeostasis in neurodegeneration. Cells. 2023;12(3):429. doi:10.3390/cells12030429. 36766772 PMC9913973

[122] Bagatini MD, Dos Santos AA, Cardoso AM, Mânica A, Reschke CR, Carvalho FB. The impact of purinergic system enzymes on noncommunicable, neurological, and degenerative diseases. J Immunol Res. 2018; 2018. 2018:1–21. doi:10.1155/2018/4892473. PMC6109496 30159340

[123] Kong G, Cao K-A L, Hannan AJ. Alterations in the gut fungal community in a mouse Model of Huntington’s disease. Microbiol Spectr. 2022;10(2):e02192–21. doi:10.1128/spectrum.02192-21. 35262396 PMC9045163

[124] Love CJ, Masson BA, Gubert C, Hannan AJ. The microbiota-gut-brain axis in Huntington’s disease. Int Rev Neurobiol Elsevier. 2022;167:141–184. 10.1016/bs.irn.2022.06.005 36427954

[125] Kong G, Cao K-A L, Judd LM, Li S, Renoir T, Hannan AJ. Microbiome profiling reveals gut dysbiosis in a transgenic mouse model of Huntington’s disease. Neurobiol Dis. 2020;135:104268. doi:10.1016/j.nbd.2018.09.001. 30194046

[126] Du G, Dong W, Yang Q, Yu X, Ma J, Gu W, Huang Y. Altered gut microbiota related to inflammatory responses in patients with Huntington’s disease. Front Immunol. 2021;11:603594. doi:10.3389/fimmu.2020.603594. 33679692 PMC7933529

[127] Clark RL, Connors BM, Stevenson DM, Hromada SE, Hamilton JJ, Amador-Noguez D, Venturelli OS. Design of synthetic human gut microbiome assembly and butyrate production. Nat Commun. 2021;12(1):3254. doi:10.1038/s41467-021-22938-y. 34059668 PMC8166853

[128] Wu S, Liu X, Jiang R, Yan X, Ling Z. Roles and mechanisms of gut microbiota in patients with Alzheimer’s disease. Front Aging Neurosci. 2021;13:650047. doi:10.3389/fnagi.2021.650047. 34122039 PMC8193064

[129] Sandhu KV, Sherwin E, Schellekens H, Stanton C, Dinan TG, Cryan JF. Feeding the microbiota-gut-brain axis: diet, microbiome, and neuropsychiatry. Transl Res. 2017;179:223–244. doi:10.1016/j.trsl.2016.10.002. 27832936

[130] Kondo T, Okada Y, Shizuya S, Yamaguchi N, Hatakeyama S, Maruyama K. Neuroimmune modulation by tryptophan derivatives in neurological and inflammatory disorders. Eur J Cell Biol. 2024;103(2):151418. doi:10.1016/j.ejcb.2024.151418. 38729083

[131] Lynch DR, Goldsberry A, Rummey C, Farmer J, Boesch S, Delatycki MB, Giunti P, Hoyle JC, Mariotti C, Mathews KD. Propensity matched comparison of omaveloxolone treatment to Friedreich ataxia natural history data. Ann Clin Transl Neurol. 2024;11(1):4–16. doi:10.1002/acn3.51897. 37691319 PMC10791025

[132] Yu J, Fan Y, Wang L, Huang Y, Xia J, Ding L, C-F W, Lu X, Ma G, Kim S. Intestinal surgery contributes to acute cerebellar ataxia through gut brain axis. Front Neurol. 2019;10:995. doi:10.3389/fneur.2019.00995. 31616359 PMC6764330

[133] Nishiwaki H, Ito M, Hamaguchi T, Maeda T, Kashihara K, Tsuboi Y, Ueyama J, Yoshida T, Hanada H, Takeuchi I. Short chain fatty acids-producing and mucin-degrading intestinal bacteria predict the progression of early Parkinson’s disease. NPJ Parkinsons Dis. 2022;8(1):65. doi:10.1038/s41531-022-00328-5. 35650236 PMC9160257

[134] Rea K, Dinan TG, Cryan JF. The microbiome: a key regulator of stress and neuroinflammation. Neurobiol Stress. 2016;4:23–33. doi:10.1016/j.ynstr.2016.03.001. 27981187 PMC5146205

[135] Borody TJ, Khoruts A. Fecal microbiota transplantation and emerging applications. Nat Rev Gastroenterol Hepatol. 2012;9(2):88–96. doi:10.1038/nrgastro.2011.244. 22183182

[136] DeFilipp Z, Bloom PP, Torres Soto M, Mansour MK, Sater MRA, Huntley MH, Turbett S, Chung RT, Chen Y-B, Hohmann E-D-RE. Drug-resistant E. coli bacteremia transmitted by fecal microbiota transplant. N Engl J Med. 2019;381(21):2043–2050. doi:10.1056/NEJMoa1910437. 31665575

[137] Cammarota G, Ianiro G, Tilg H, Rajilić-Stojanović M, Kump P, Satokari R, Sokol H, Arkkila P, Pintus C, Hart A. European consensus conference on faecal microbiota transplantation in clinical practice. Gut; Vol. 66. 2017. p. 569–580. doi:10.1136/gutjnl-2016-313017. PMC5529972 28087657

[138] Borre YE, Gw O, Clarke G, Stanton C, Dinan TG, Cryan JF. Microbiota and neurodevelopmental windows: implications for brain disorders. Trends Mol Med. 2014;20(9):509–518. doi:10.1016/j.molmed.2014.05.002. 24956966

[139] Hsiao EY, Sw M, Hsien S, Sharon G, Hyde ER, McCue T, Codelli JA, Chow J, Reisman SE, Petrosino JF. Microbiota modulate behavioral and physiological abnormalities associated with neurodevelopmental disorders. Cell. 2013;155(7):1451–1463. doi:10.1016/j.cell.2013.11.024. 24315484 PMC3897394

[140] Anderson G. A more holistic perspective of Alzheimer’s disease: roles of gut microbiome, adipocytes, HPA axis, Melatonergic Pathway and astrocyte mitochondria in the emergence of autoimmunity. Front Biosci-Landmark. 2023;28:355. doi:10.31083/j.fbl2812355. 38179773

